# Supplementary material for: Adipose tissue-derived neurotrophic factor 3 regulates sympathetic innervation and thermogenesis in adipose tissue
Source: Nat Commun. 2021 Sep 10;12:5362. doi: 10.1038/s41467-021-25766-2 (PMC8433218; doi:10.1038/s41467-021-25766-2)
Supplement: Supplementary file 1 — Supplementary Information [file 41467_2021_25766_MOESM1_ESM.pdf]

Supplemental Tables, Figures and Figure Legends

Adipose tissue-derived neurotrophic factor 3 regulates sympathetic innervation and thermogenesis in adipose tissue

Xin Cui[1], Rui Wu[1], Qiang Cao[1], Fenfen Li[1], Lizhi Fu[1], Ke Li[1], Jia Jing[1], Liqing Yu[2], Gary Schwartz[3], Huidong Shi[4], Bingzhong Xue[1]* and Hang Shi[1]*

[1]Department of Biology, Georgia State University, Atlanta, GA 30303, [2]Department of Medicine, University of Maryland School of Medicine, Baltimore, MD 21201; [3]Department of Medicine, Albert Einstein College of Medicine, Bronx, NY 10461; [4]Department of Biochemistry and Molecular Biology, Augusta University, Augusta, GA 30912.

Corresponding authors:

Hang Shi

Department of Biology, Georgia State University, 24 Peachtree Center Avenue, Atlanta, GA 30303, USA

E-mail: hshi3@gsu.edu

Phone: 404-413-5799

Bingzhong Xue

Department of Biology, Georgia State University, 24 Peachtree Center Avenue, Atlanta, GA 30303, USA

E-mail: bxue@gsu.edu

Phone: 404-413-5747

Running Title: NT-3 regulates sympathetic innervation in adipose tissue.

Supplemental Table 1. Antibodies used in Immunoblotting, IF and IHC

Antibody	Company	Catalog #	Application
**UCP1**	Abcam	ab23841	WB (1:1000)
**UCP1**	Abcam	Ab10983	IHC (1:500)
**TH**	Millipore	Ab152	WB (1:1000), IF (1:500, 1:1000 for whole mount clearing)
**pHSL**	Cell Signaling Technology	4126S	WB (1:1000)
**HSL**	Cell Signaling Technology	4107S	WB (1:1000)
**NT-3**	R&D Systems	AF-267-NA	WB (1:1000)
**cFos**	Millipore	ABE457	IF (1:500)
**GFP**	Aves labs	GFP-1010	IF (1:500)
**βIII-tubulin, Alexa Fluor® 488 Conjugate**	Millipore	AB15708A4	IF (1:400)
**α-Tubulin**	Advanced BioChemicals	ABCENT4777	WB (1:1000)
**Biotin-SP (long spacer) AffiniPure Donkey Anti-Rabbit IgG (H+L)**	Jackson ImmunoResearch	711-065-152	IHC (1:500)
**Cy™3 AffiniPure Donkey Anti-Rabbit IgG (H+L)**	Jackson ImmunoResearch	711-165-152	IF (1:500, 1:2000 for whole mount clearing)
**Alexa Fluor® 488 AffiniPure Donkey Anti-Chicken IgY (IgG) (H+L)**	Jackson ImmunoResearch	703-545-155	IF (1:500)
**Donkey anti-Goat IgG (H+L) Cross-Adsorbed Secondary Antibody, Alexa Fluor 680**	Invitrogen	A21084	WB (1: 5000)
**Goat anti-Rabbit IgG (H+L) Highly Cross-Adsorbed Secondary Antibody, Alexa Fluor 680**	Invitrogen	A21109	WB (1:5000)

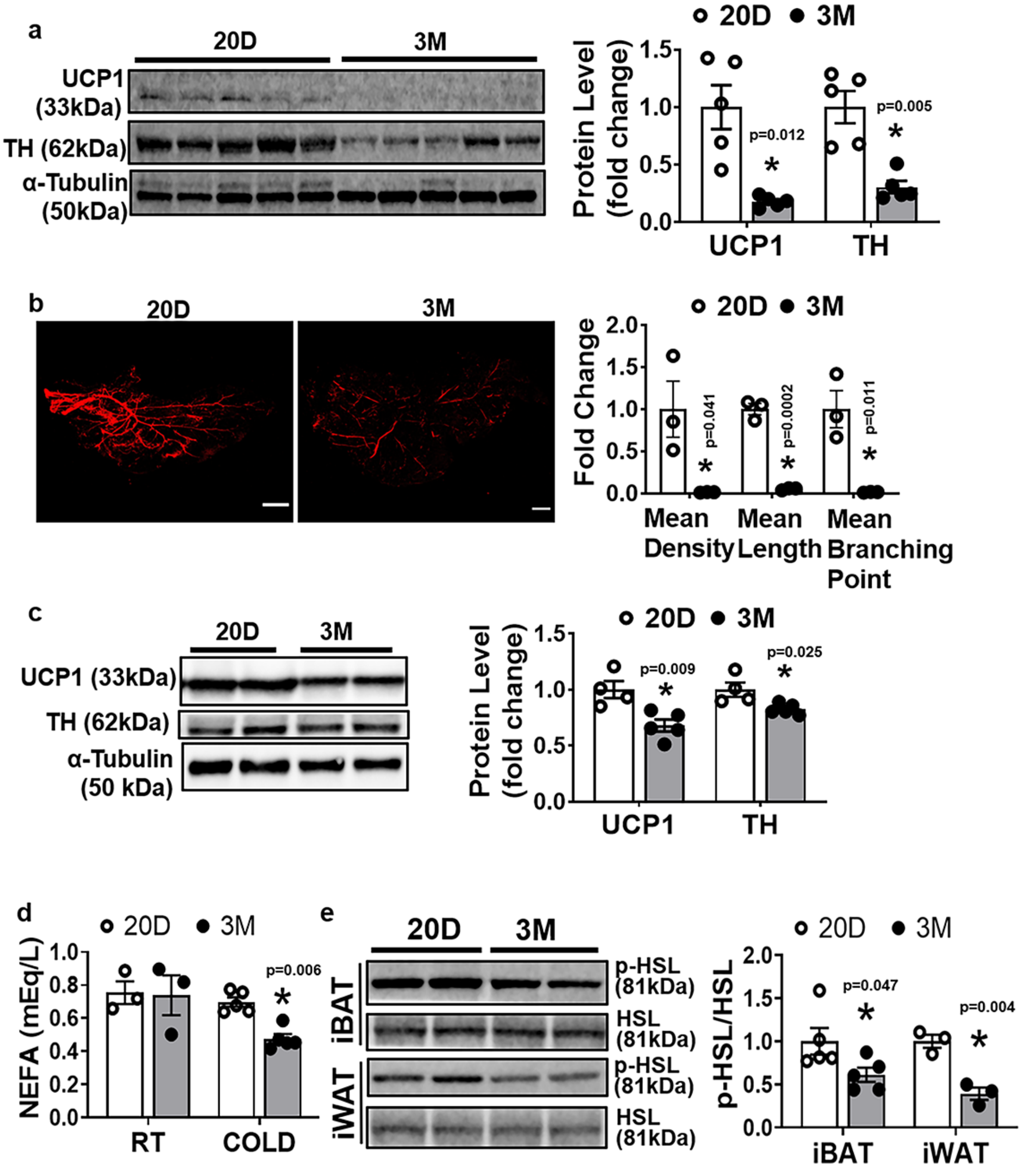

Supplemental Figure 1. Adipose-derived neurotrophic factor NT-3 correlates with UCP1 expression and sympathetic innervation in adipose tissue.

(**a**) Immunoblotting (left panel) and quantitation (right panel) of UCP1 and TH protein in eWAT of 20 days old (20D) postnatal pups and 3-month-old (3M) adult mice (n=5/group, *indicates statistical significance between 20D and 3M with unpaired two-tailed t-test).

(**b**) Representative images of TH-positive sympathetic nerve innervation in eWAT (left, from 3 replicate animals/group, scale bar=2000μm) and quantitation of mean nerve fiber density, mean nerve fiber length, and mean branching points normalized to total adipose tissue area in eWAT (right panel, n=3/group, *indicates statistical significance between 20D and 3M with unpaired two-tailed t-test) of 20 days old postnatal pups and 3-month-old adult mice.

(**c**) Immunoblotting (left) and quantitation (right panel) of UCP1 and TH protein in iBAT of 20 days old postnatal pups and 3-month-old adult mice (20D n=4, 3M n=5, *indicates statistical significance between 20D and 3M with unpaired two-tailed t-test).

(**d**) serum NEFA levels in 20 days old postnatal pups and 3-month-old adult mice at room temperature (RT) and after a 6-hour 10°C cold challenge (RT n=3, cold n=5, *indicates statistical significance between 20D and 3M with unpaired two-tailed t-test).

(**e**) Total and phosphorylated hormone sensitive lipase (HSL and pHSL, respectively) in iBAT and iWAT of 20 days old postnatal pups and 3-month-old adult mice after the cold challenge (iBAT n=5/group, iWAT n=3/group, *indicates statistical significance between 20D and 3M with unpaired two-tailed t-test).

All data are expressed as mean ± SEM.

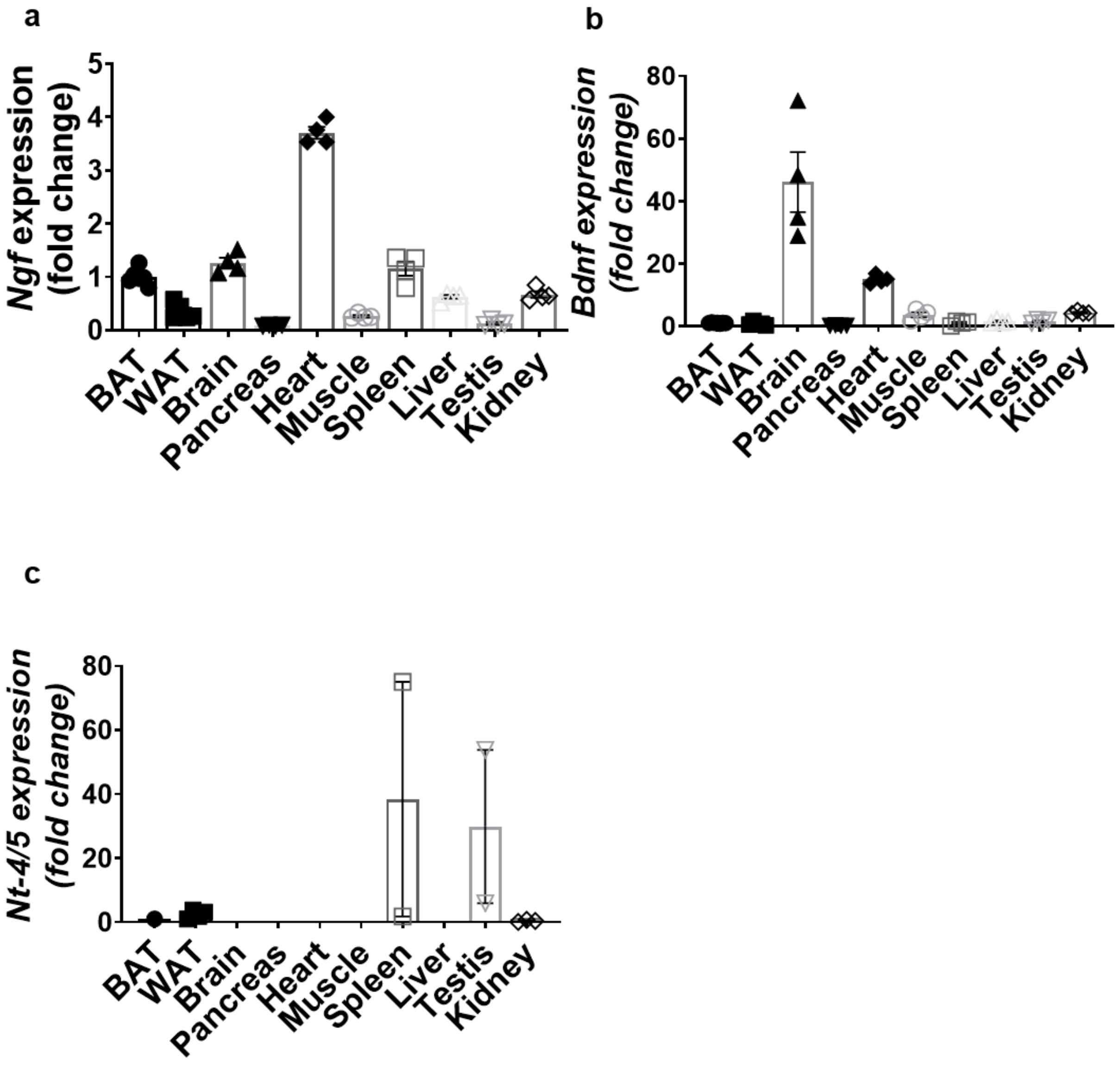

Supplemental Figure 2. Tissue distribution of neurotrophic factors NGF (**a**), BDNF (**b**) and NT4/5 (**c**) (BAT n=6, WAT n=5, others n=4). For (**c**), *Nt-4/5* expression was undetectable in Brain, Pancreas, Heart, Muscle and Liver; two out of four samples in Spleen and Testis, five out of six samples in BAT, and one out of four samples in Kidney were undetectable.

All data are expressed as mean ± SEM.

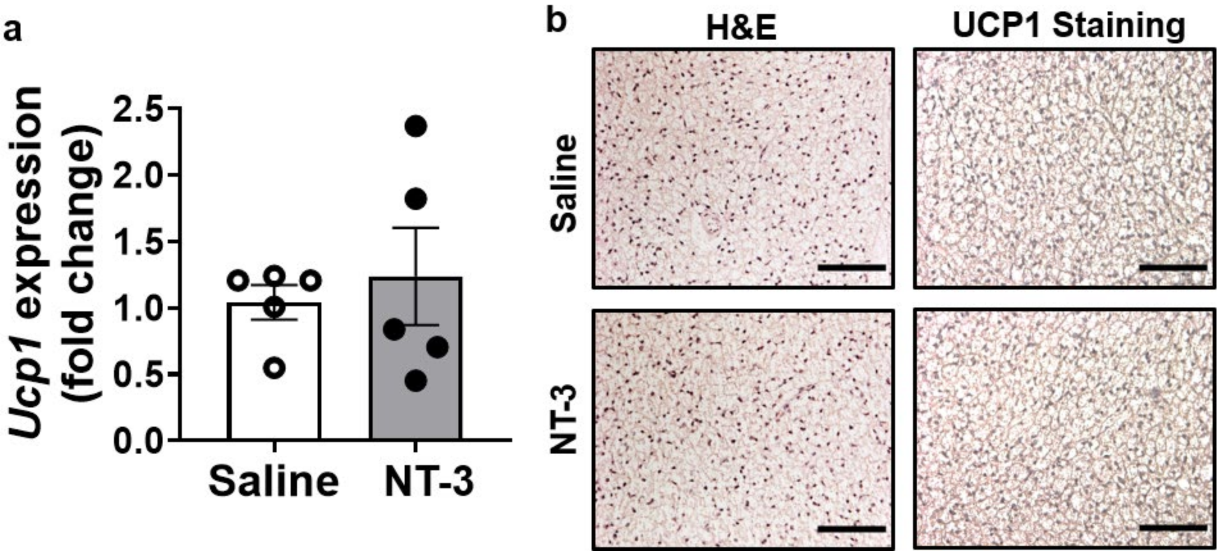

Supplemental Figure 3. *Ucp1* expression (**a,** n=5/group) and H&E and representative UCP1 immunostaining (**b**, from 3 replicate animals/group, scale bar=75µm) in iBAT of 20 days old postnatal pups with daily intraperitoneal (ip) NT-3 (50 µg/kg) injection for 10 days.

All data are expressed as mean ± SEM.

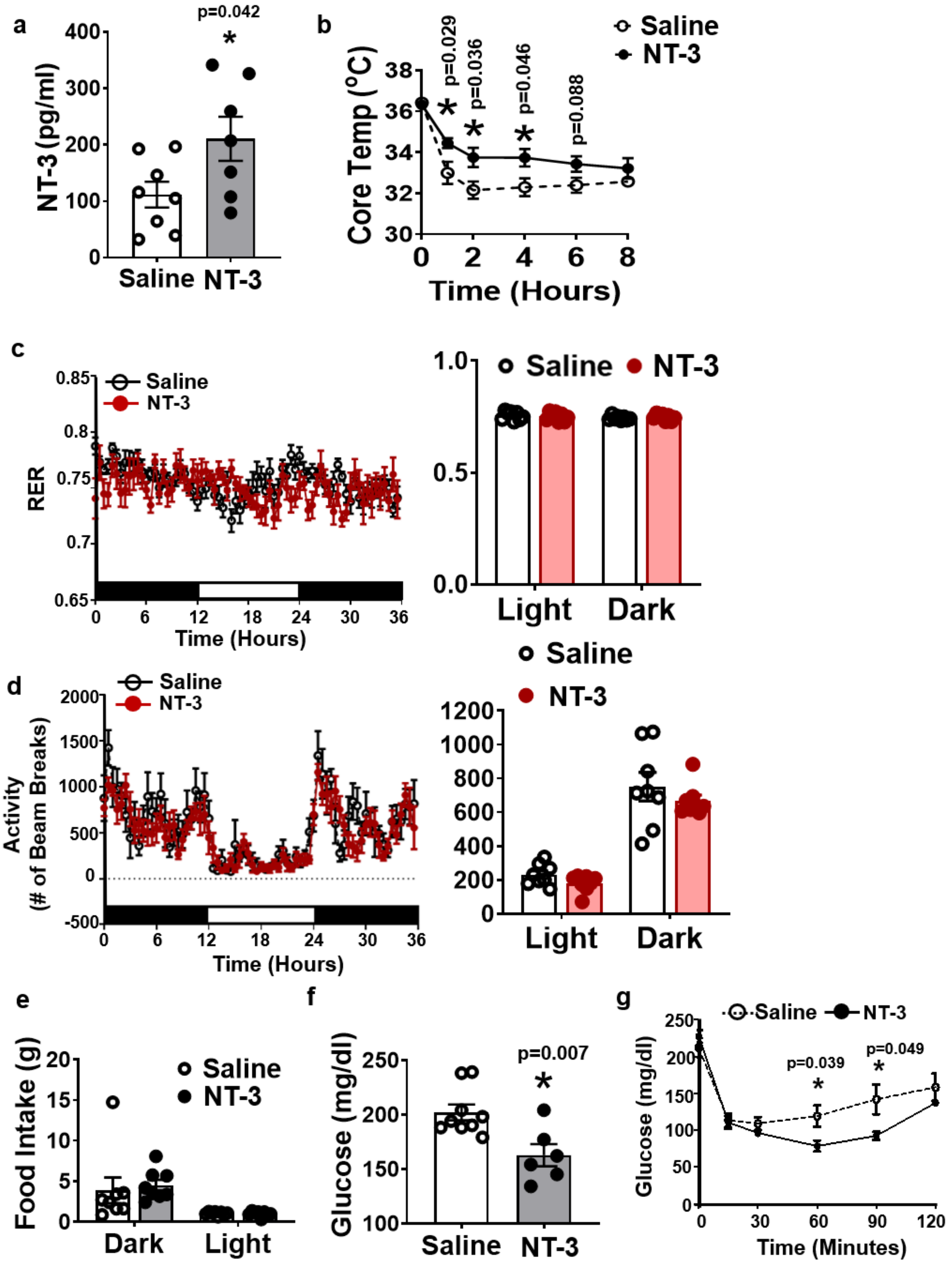

a
p=0.042
NT-3 (pg/ml)
Saline NT-3
b
Saline
NT-3
p=0.029
p=0.036
p=0.046
p=0.088
Core Temp (°C)
Time (Hours)
c
Saline
NT-3
RER
Time (Hours)
Light Dark
d
Saline
NT-3
Activity (# of Beam Breaks)
Time (Hours)
Light Dark
e
Food Intake (g)
Saline
NT-3
Dark Light
f
Glucose (mg/dl)
p=0.007
Saline NT-3
g
Saline
NT-3
Glucose (mg/dl)
p=0.039
p=0.049
Time (Minutes)

Supplemental Figure 4. Metabolic characterization of NT-3-injected mice with 5°C cold challenge or fed HFD.

(**a**) Serum NT-3 levels in saline and NT-3-injected mice (saline n=8, NT-3 n=7, *indicates statistical significance between saline and NT-3 treatments with unpaired two-tailed t-test).

(**b**) Core body temperature in saline- and NT-3-injected mice subjected to an acute 5°C cold exposure (Saline n=5, NT-3 n=6, *indicates statistical significance between saline and NT-3 treatments with unpaired two-tailed t-test).

(**c**-**g**) Respiratory exchange rate (RER) (**c**, n=8/group), Locomotor activity (**d**, n=8/group), Food intake (**e**, n=8/group), Fed glucose levels (**f**) (saline n=9, NT-3 n=6, *indicates statistical significance between saline and NT-3 treatments with unpaired two-tailed t-test), and Insulin tolerance test (ITT) (**g**) (saline n=7, NT-3 n=6, *indicates statistical significance between saline and NT-3 treatments with unpaired two-tailed t-test) in C57BL/6J mice injected with NT-3 with HFD feeding.

All data are expressed as mean ± SEM.

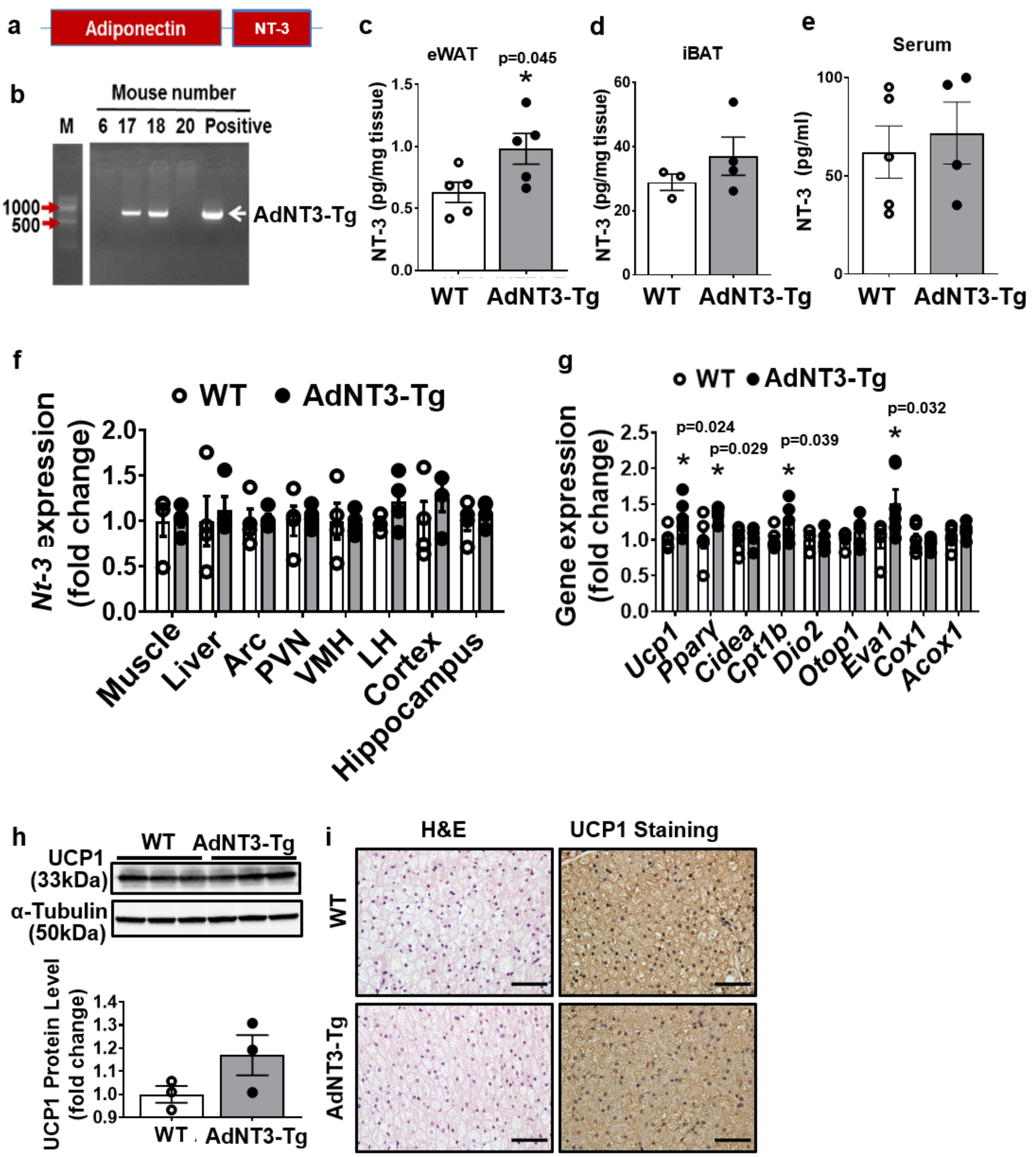

Supplemental Figure 5. Generation and metabolic characterization of mice with adipocyte-specific overexpression of NT-3 (AdNT3-Tg).

(**a**) Schematic illustration of adiponectin-NT-3 overexpressing construct.

(**b**) Genotyping identification of positive AdNT3-Tg mice.

(**c**)-(**e**) NT-3 protein levels in eWAT (**c**, n=5/group, *indicates statistical significance between WT and AdNT3-Tg with unpaired two-tailed t-test), iBAT (**d**, WT n=3 AdNT3Tg n=4) and serum (**e**, WT n=5, AdNT3-Tg n=4) of WT and AdNT3-Tg mice as measured by ELISA.

(**f**) *Nt-3* expression in various tissues, including gastrocnemius muscle, liver, arcuate (ARC), paraventricular (PVN), ventromedial (VMH) and lateral (LH) hypothalamus, cortex and hippocampus (n=4/group).

(**g**)-(**i**) *Ucp1* and other thermogenic gene expression (**g**, WT n=6 AdNTF3 n=5, *indicates statistical significance between WT and AdNT3-Tg with unpaired two-tailed t-test), UCP1 protein levels (**h**, n=3/group) and representative H&E and UCP1 immunostaining in iBAT (**i**, from 3 replicate animals/group, scale bar=75μm) of WT and AdNT3-Tg mice subjected to a 7-day cold exposure.

All data are expressed as mean ± SEM.

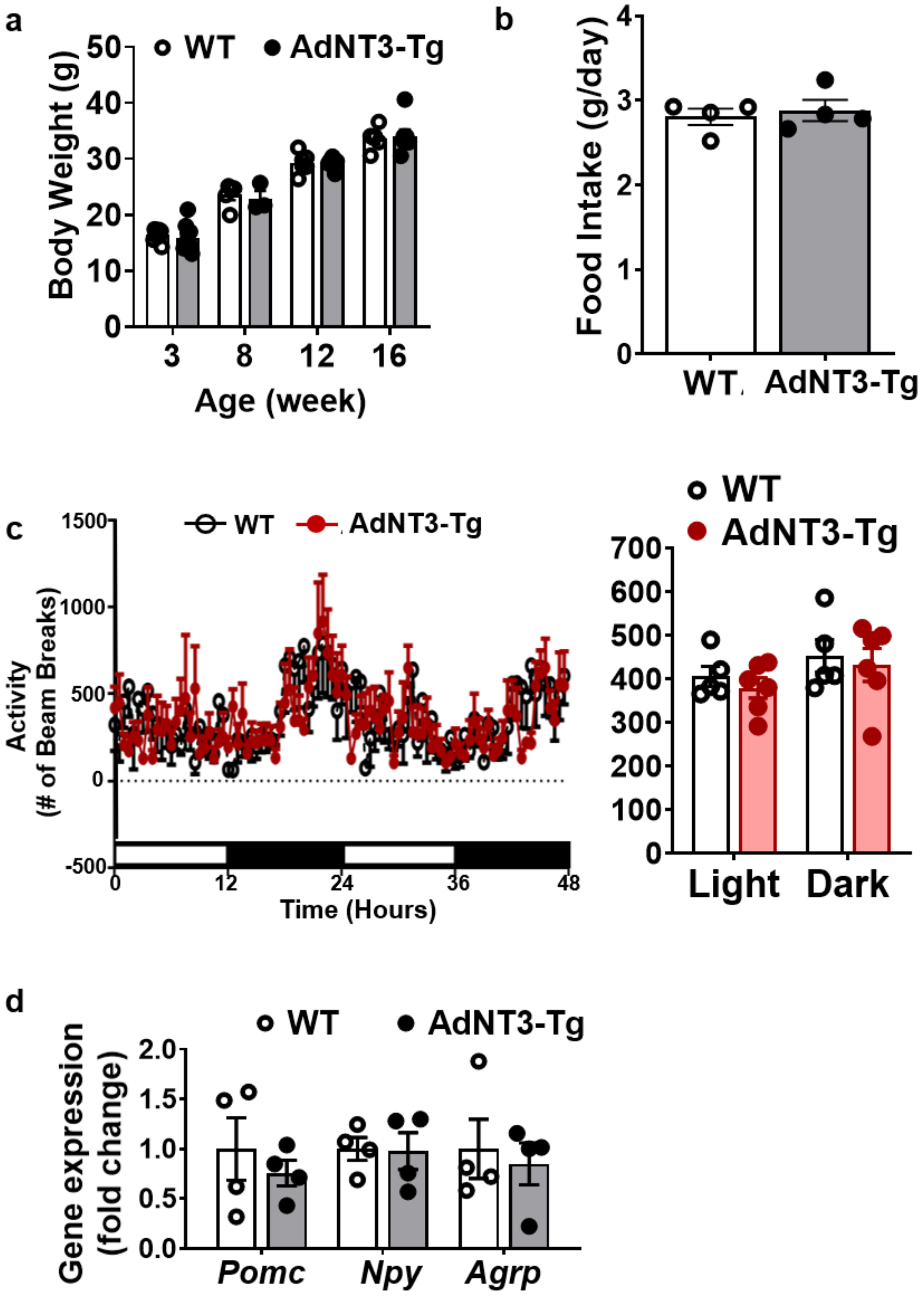

Supplemental Figure 6. Metabolic characterization of WT and AdNT3-Tg mice fed regular chow or HFD.

(**a**) Body weight of AdNT3-Tg and WT mice fed with regular chow diet (3 weeks=9/group, 8 weeks WT=5, AdNT3Tg=3, 12 weeks=6/group, 16 weeks=6/group).

(**b**-**c**) Food intake (**b**, n=4/group), Locomotor activity (**c**, WT n=5, AdNT3-Tg n=6) of WT and AdNT3-Tg mice fed HFD.

(**d**) Expression of the neuropeptides pro-opiomelanocortin (*Pomc*), neuropeptide Y (*Npy*), and agouti related neuropeptide (*Agrp*) in arcuate hypothalamus (ARC) of AdNT3-Tg and WT littermates fed HFD (n=4/group).

All data are expressed as mean ± SEM.

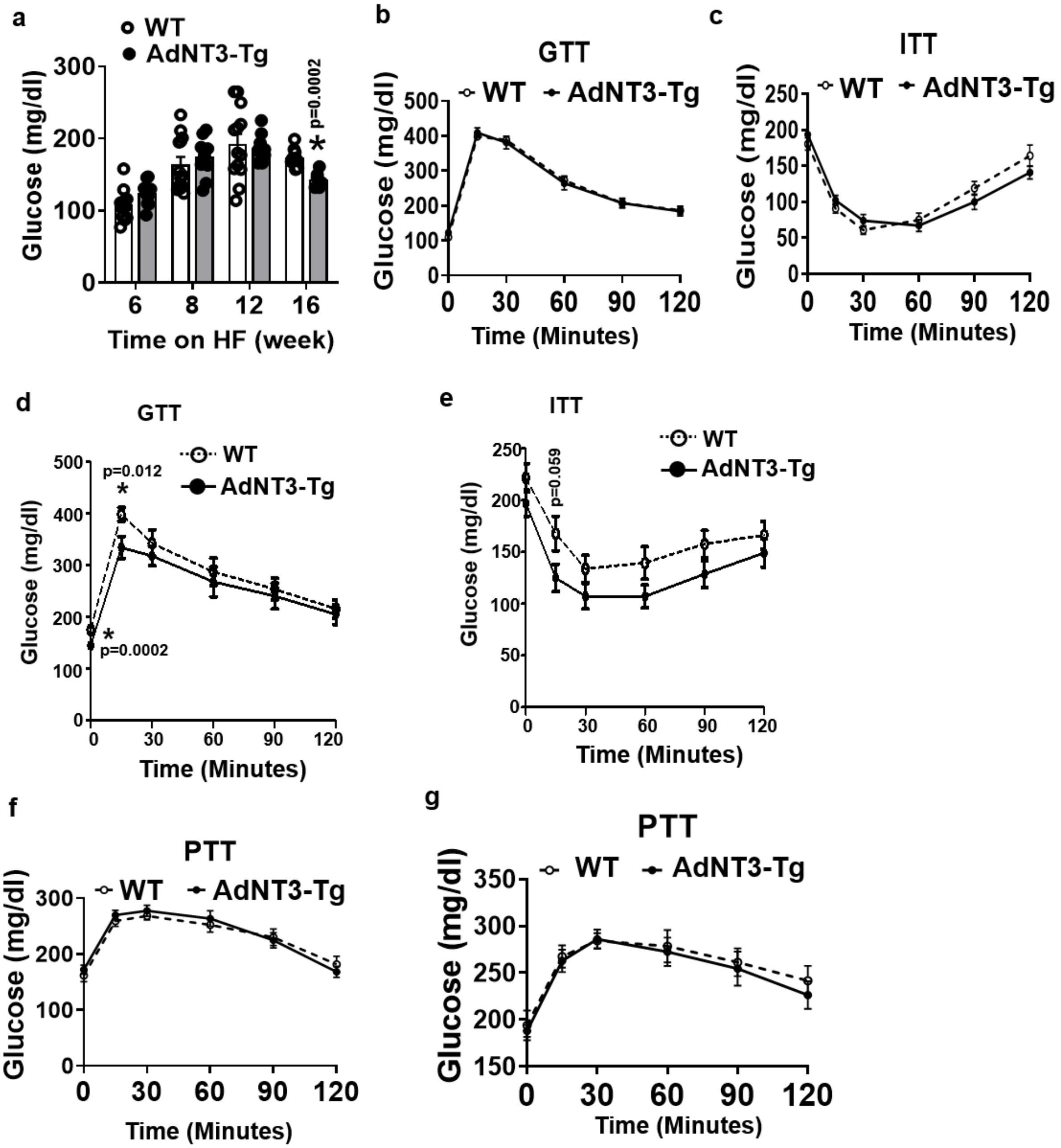

Supplemental Figure 7. Regulation of glucose homeostasis in AdNT3-Tg and WT mice fed HFD.

(**a**) Fasting glucose levels in HFD-fed AdNT3-Tg and WT mice measured at various time points. For 6 weeks, WT=13, AdNT3-Tg=12; 8 weeks, WT=13, AdNT3-Tg=11; 12 weeks, n=11/group; 16 weeks, WT=9, AdNT3-Tg=7. *indicates statistical significance between WT and AdNT3-Tg with unpaired two-tailed t-test.

(**b**-**c**) GTT (**b**, WT=13, AdNT3-Tg=12) and ITT (**c**, WT=7, AdNT3-Tg=6) in AdNT3-Tg and WT mice measured at 6-7 weeks of HFD feeding.

(**d**-**e**) GTT (**d**, WT=9, AdNT3-Tg=7, *indicates statistical significance between WT and AdNT3-Tg with unpaired two-tailed t-test) and ITT (**e**, WT=8, AdNT3-Tg=9, *indicates statistical significance between WT and AdNT3-Tgwith unpaired two-tailed t-test) in AdNT3-Tg and WT mice measured at 15-16 weeks of HFD feeding.

(**f**-**g**) PTT in AdNT3-Tg and WT mice measured at 8 weeks (**f**, WT=13, AdNT3-Tg=12) and 14 weeks (**g**, WT=11, AdNT3-Tg=12) of HFD feeding.

All data are expressed as mean ± SEM.

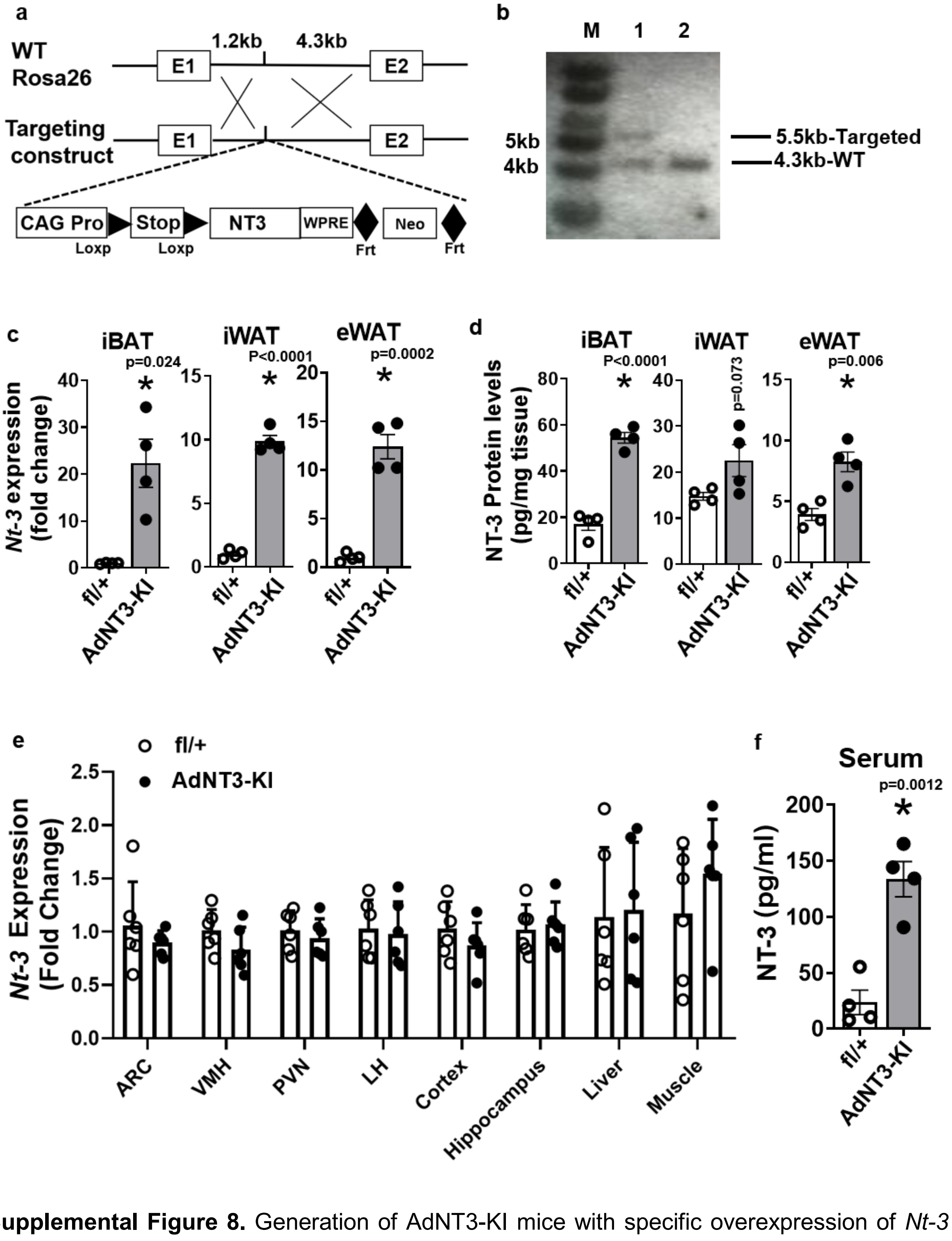

Supplemental Figure 8. Generation of AdNT3-KI mice with specific overexpression of *Nt-3* in adipocytes.

(**a**) Schematic diagram of the gene targeting strategy to insert *Nt-3* transgene into the *Rosa26* locus. CAG Pro: CAG promoter; Stop: transcriptional blocker; WPRE: woodchuck hepatitis virus

posttranslational regulatory element; FRT: flippase (*Flp*) recognition target; Neo: neomycin selecting cassette.

(**b**) Representative Southern blot for screening of homologous recombination of ES clones (from 2 independent experiments).

(**c**)-(**d**) *NT-3* mRNA (**c**, n=4/group, *indicates statistical significance between fl/+ and AdNT3-KI with unpaired tailed t-test) and protein levels (**d**, n=4/group, *indicates statistical significance with unpaired tailed t-test) in fat tissue of 8 weeks old AdNT3-KI and the control fl/+ mice.

(**e**) *Nt-3* expression in various tissues, including gastrocnemius muscle, liver, arcuate (ARC), paraventricular (PVN), ventromedial (VMH) and lateral (LH) hypothalamus, cortex and hippocampus (n=6/group).

(**f**) NT-3 protein levels in serum of AdNT3-KI and control fl/+ littermates (n=4/group, *indicates statistical significance between fl/+ and AdNT3-KI with unpaired two-tailed t-test).

All data are expressed as mean ± SEM.

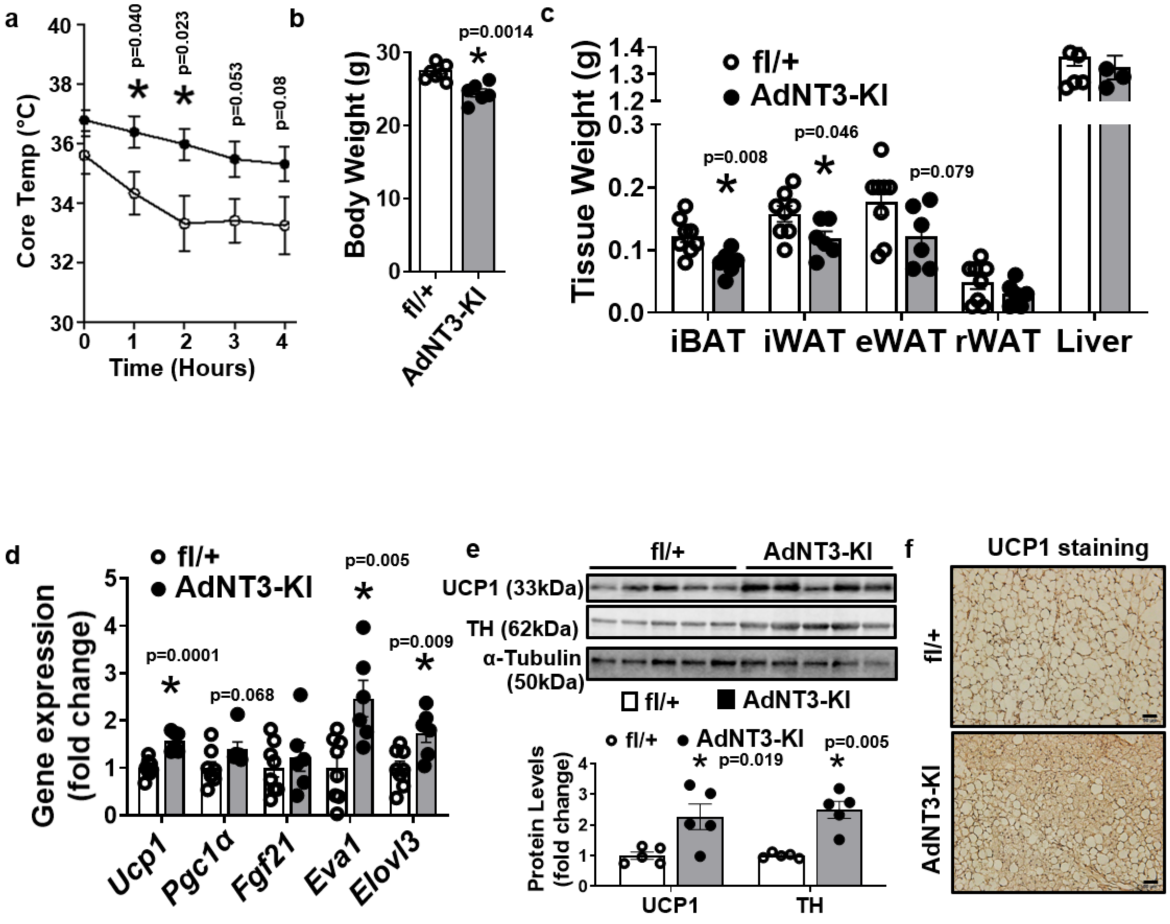

Supplemental Figure 9. Metabolic characterization of fl/+ and AdNT3-KI mice challenged with 5°C cold exposure.

(**a**)-(**f**) Body temperature (**a**, fl/+ n=5 AdNT3-KI n=7, *indicates statistical significance between fl/+ and AdNT3-KI with unpaired two-tailed t-test), Body weight (**b**, fl/+ n=8 AdNT3-KI n=6, *indicates statistical significance between fl/+ and AdNT3-KI with unpaired two-tailed t-test), Fat pad mass (**c**, fl/+ n=8 AdNT3-KI n=6, *indicates statistical significance between fl/+ and AdNT3-KI with unpaired two-tailed t-test), *Ucp1* and other thermogenic gene expression in iWAT (**d,** fl/+ n=8 AdNT3-KI n=6, fl/+ n=8 AdNT3-KI n=6, *indicates statistical significance between fl/+ and AdNT3-KI with unpaired two-tailed t-test), UCP1 and TH protein levels in iWAT (**e**, n=5/group, *indicates statistical significance between fl/+ and AdNT3-KI with unpaired two-tailed t-test) and representative UCP1 immunostaining image showing UCP1-positive beige adipocytes in iWAT (**f**, from 3 replicate animals/group, scale bar=50μm) in 8 weeks old fl/+ and AdNT3-KI mice in response to a 7-day cold challenge.

All data are expressed as mean ± SEM. *p<0.05.

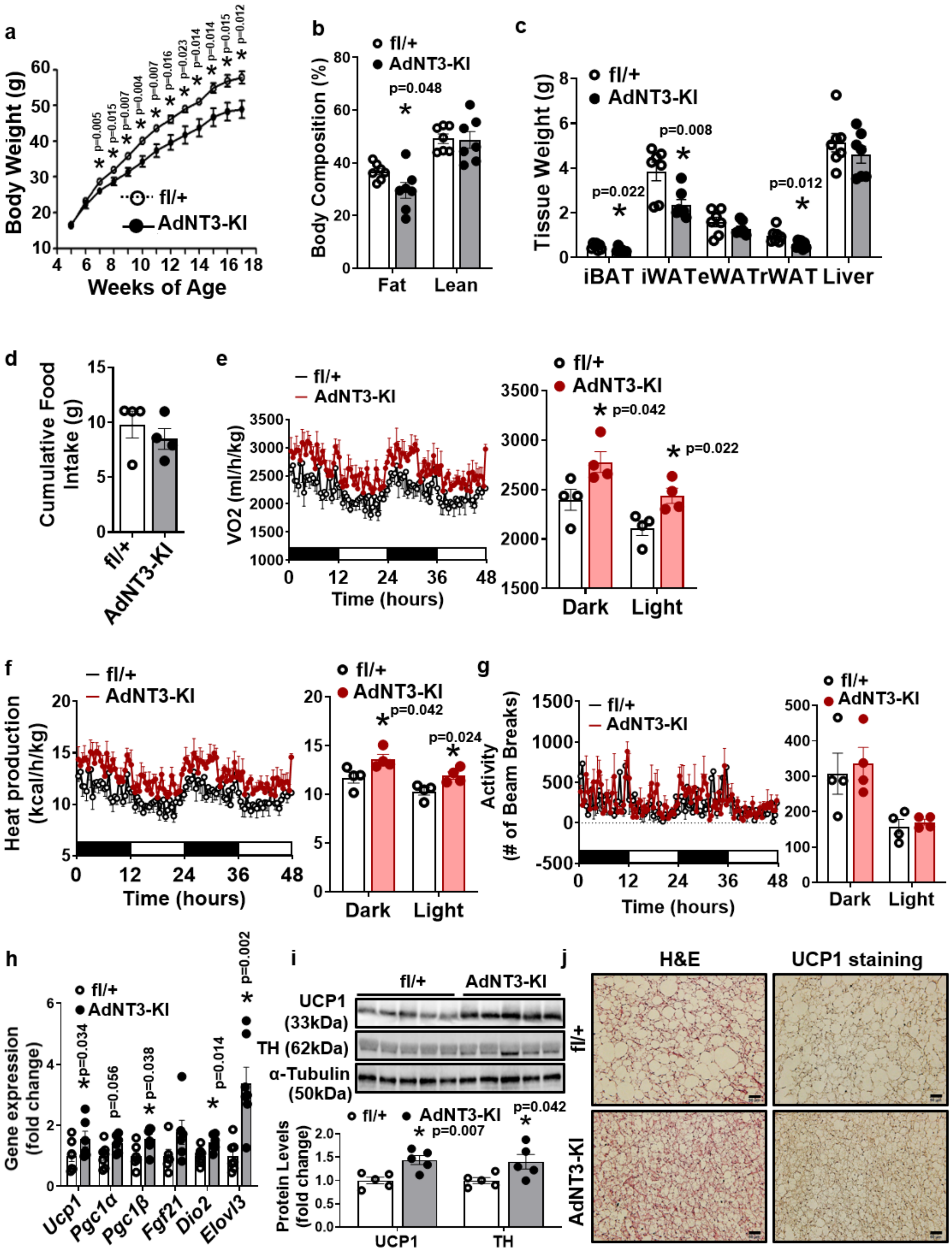

a
Body Weight (g)
Weeks of Age
fl/+
AdNT3-KI
p=0.005
p=0.015
p=0.007
p=0.004
p=0.007
p=0.016
p=0.023
p=0.014
p=0.014
p=0.015
p=0.012
b
Body Composition (%)
fl/+
AdNT3-KI
p=0.048
Fat
Lean
c
Tissue Weight (g)
fl/+
AdNT3-KI
p=0.022
p=0.008
p=0.012
iBAT iWAT eWAT rWAT Liver
d
Cumulative Food Intake (g)
fl/+
AdNT3-KI
e
VO2 (ml/h/kg)
Time (hours)
p=0.042
p=0.022
Dark
Light
f
Heat production (kcal/h/kg)
Time (hours)
p=0.042
p=0.024
Dark
Light
g
Activity (# of Beam Breaks)
Time (hours)
Dark
Light
h
Gene expression (fold change)
p=0.034
p=0.056
p=0.038
p=0.014
p=0.002
Ucp1
Pgc1α
Pgc1β
Fgf21
Dio2
Elovl3
i
UCP1 (33kDa)
TH (62kDa)
α-Tubulin (50kDa)
Protein Levels (fold change)
p=0.007
p=0.042
UCP1
TH
j
H&E
UCP1 staining
fl/+
AdNT3-KI

Supplemental Figure 10. Metabolic characterization of fl/+ and AdNT3-KI mice fed HFD.

(**a-j**) Body weight (**a**, n=7/group, *indicates statistical significance between fl/+ and AdNT3-KI with unpaired two-tailed t-test), Body composition (**b**, n=7/group, *indicates statistical significance between fl/+ and AdNT3-KI with unpaired two-tailed t-test), Tissue weight (**c**, n=7/group, *indicates statistical significance between fl/+ and AdNT3-KI with unpaired two-tailed t-test), Cumulative food intake over 6 days (**d**, n=4/group), Oxygen consumption (**e**, n=4/group, *indicates statistical significance between fl/+ and AdNT3-KI with unpaired two-tailed t-test), Heat production (**f**, n=4/group, *indicates statistical significance between fl/+ and AdNT3-KI with unpaired two-tailed t-test), Locomotor activity (**g**, n=4/group), *Ucp1* and other gene expression in iBAT (**h**, fl/+=7, except for *Pgc1β* and *Fgf21*, where n=6, AdNT3KI=6, *indicates statistical significance between fl/+ and AdNT3-KI with unpaired two-tailed t-test), UCP1 and TH protein levels in iBAT (**i**, n=5/group, *indicates statistical significance between fl/+ and AdNT3-KI with unpaired two-tailed t-test), Representative H&E and UCP1 immunostaining in iBAT (**j**, from 3 replicate animals/group, scale bar=50μm).

All data are expressed as mean ± SEM.

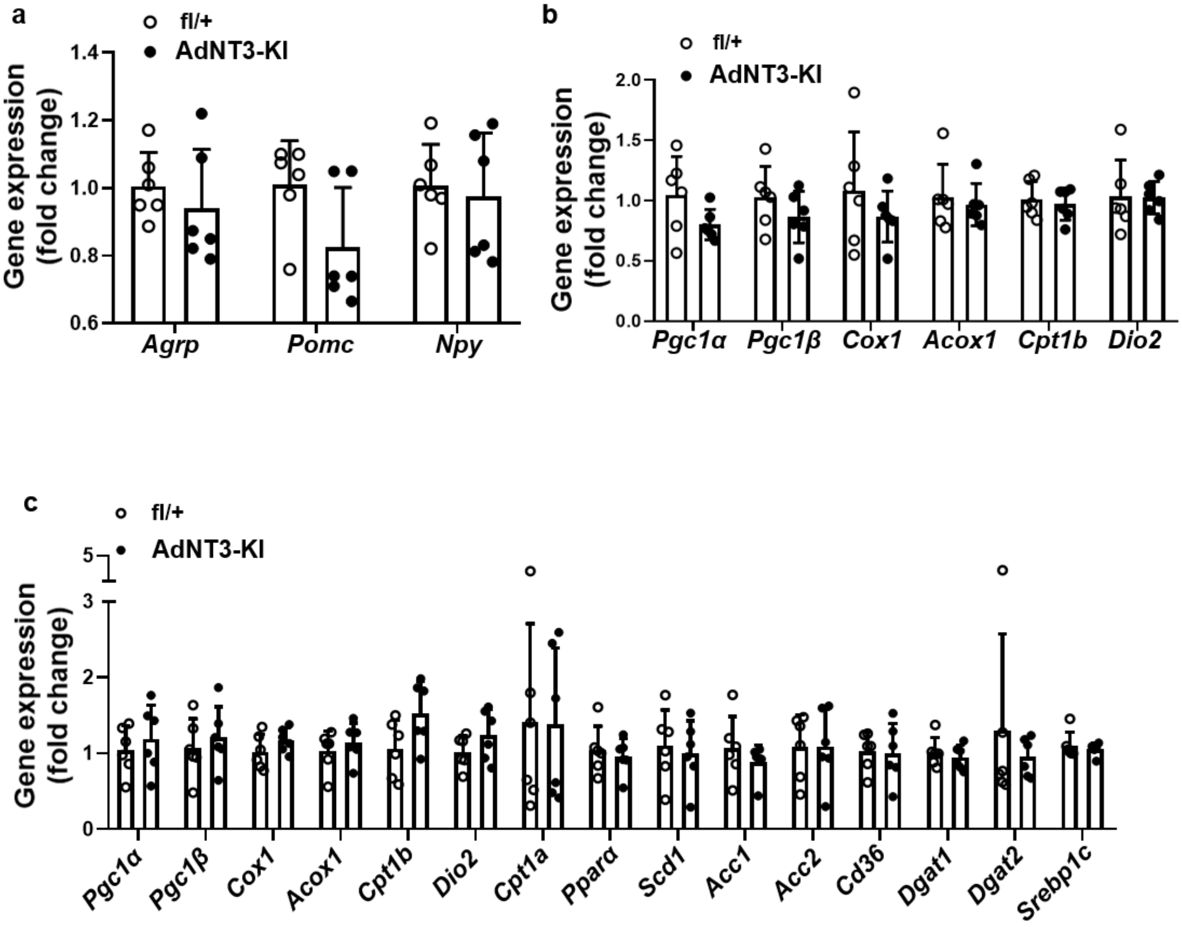

Supplemental Figure 11. Gene expression profiling in AdNT3-KI and fl/+ mice fed HFD diet.

(**a**-**c**) Gene expression measurements in arcuate hypothalamus (**a**, n=6/group), gastrocnemius muscle (**b**, n=6/group) and liver (**c**, n=6/group) of AdNT3-KI and fl/+ mice fed HFD diet. n=6/group.

All data are expressed as mean ± SEM.

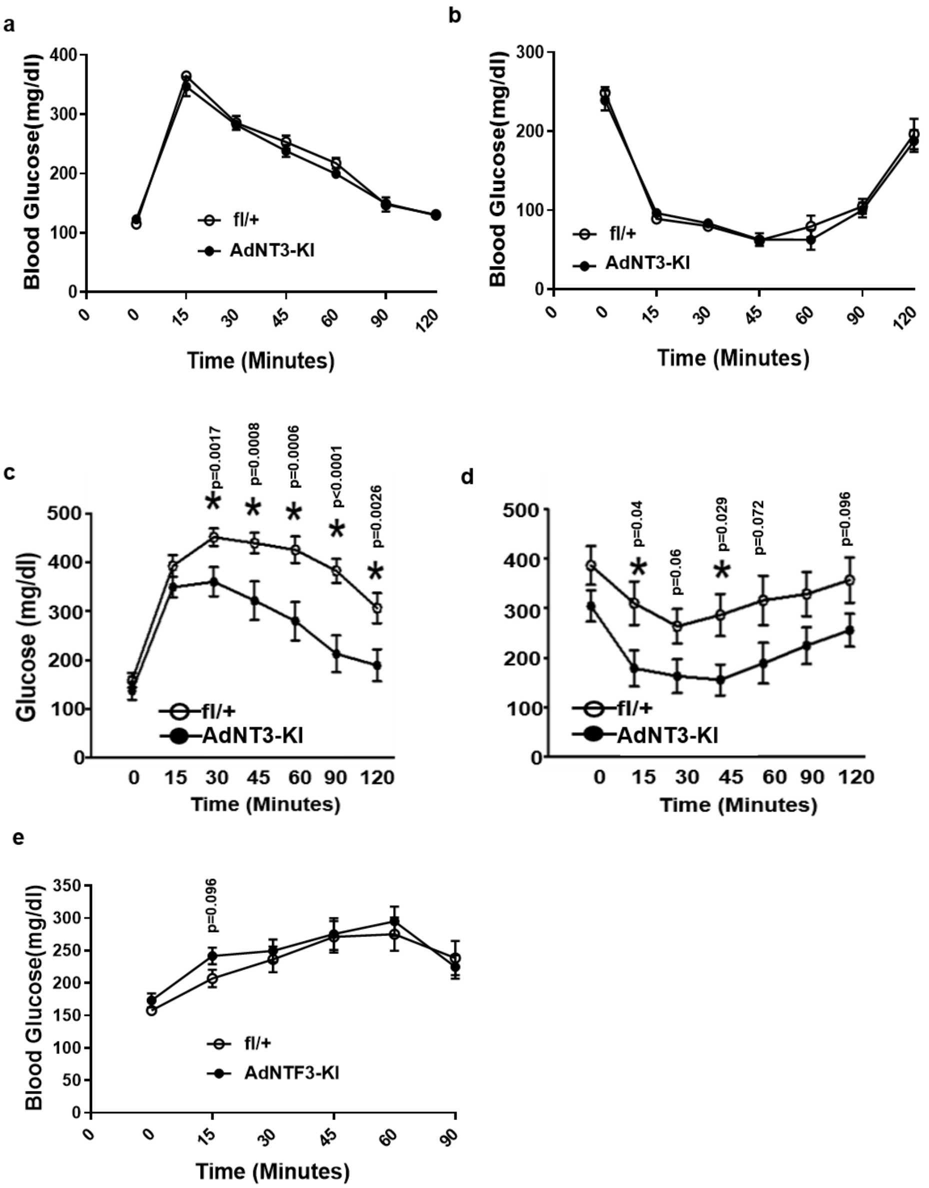

Supplemental Figure 12. Regulation of glucose homeostasis in WT and AdNT3-KI fed HFD.

(**a-b**) GTT (**a**, n=6/group) and ITT (**b**, n=6/group) in HFD-fed WT and AdNT3-KI mice measured at 3-4 weeks of HFD feeding, respectively.

(**c-d**) GTT (**c**, n=6/group, *indicates statistical significance between fl/+ and AdNT3-KI with unpaired two-tailed t-test) and ITT (**d**, n=7/group, *indicates statistical significance between fl/+ and AdNT3-KI with unpaired two-tailed t-test) in HFD-fed WT and AdNT3-KI mice measured at 15-16 weeks of HFD feeding, respectively.

(**e**) PTT in HFD-fed WT and AdNT3-KI mice measured at 14 weeks of HFD feeding (n=6/group).

All data are expressed as mean ± SEM.

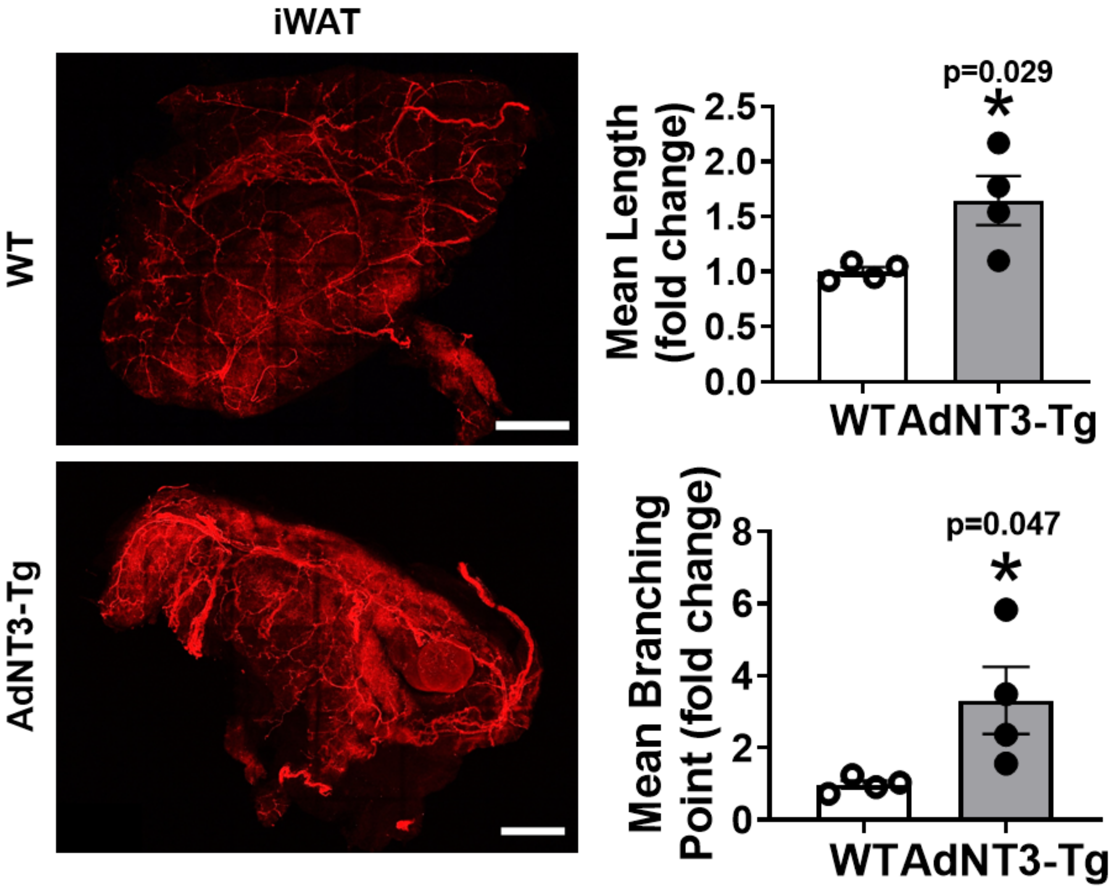

Supplemental Figure 13. Representative images of iWAT TH-positive sympathetic nerve innervation (left panel, from 4 replicate animals/group, scale bar=2000μm) and quantitation of mean nerve fiber length and mean nerve fiber branching points normalized to total adipose tissue area (right panel, n=4/group, *indicates statistical significance between WT and AdNT3-Tg with unpaired two-tailed t-test) in 2-month-old WT and AdNT3-Tg mice housed at room temperature.

All data are expressed as mean ± SEM.

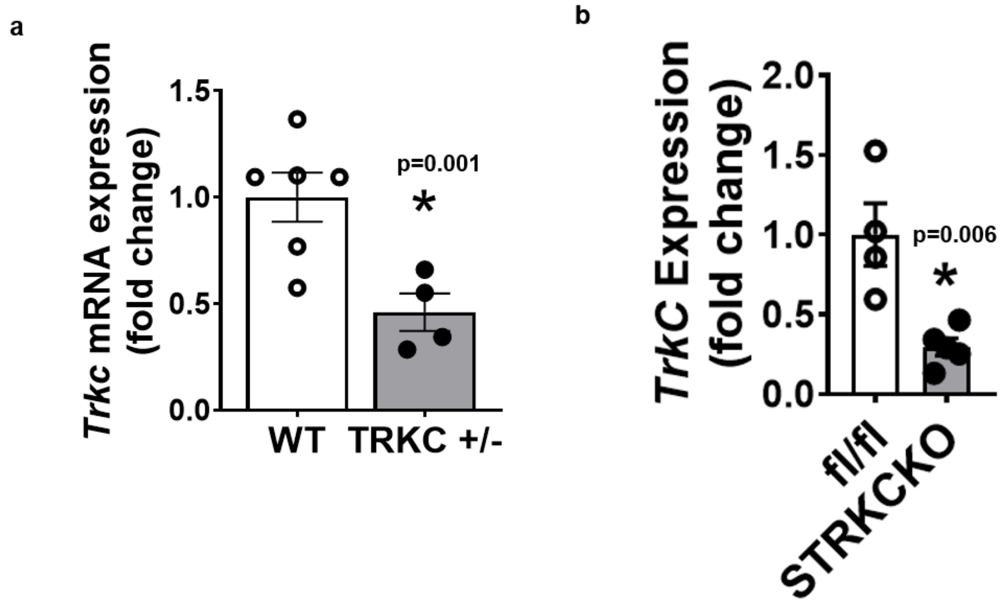

Supplemental Figure 14. *Trkc* expression in sympathetic ganglia of WT and TRKC+/- mice (**a**, WT=6, TRKC+/-=4, *indicates statistical significance between WT and TRKC+/- with unpaired two-tailed t-test) and fl/fl and sympathetic TRKC knockout (STRKCKO) mice (**b**, fl/fl=4 STRKCKO=5, *indicates statistical significance between fl/fl and STRKCKO with unpaired two-tailed t-test).

All data are expressed as mean ± SEM.

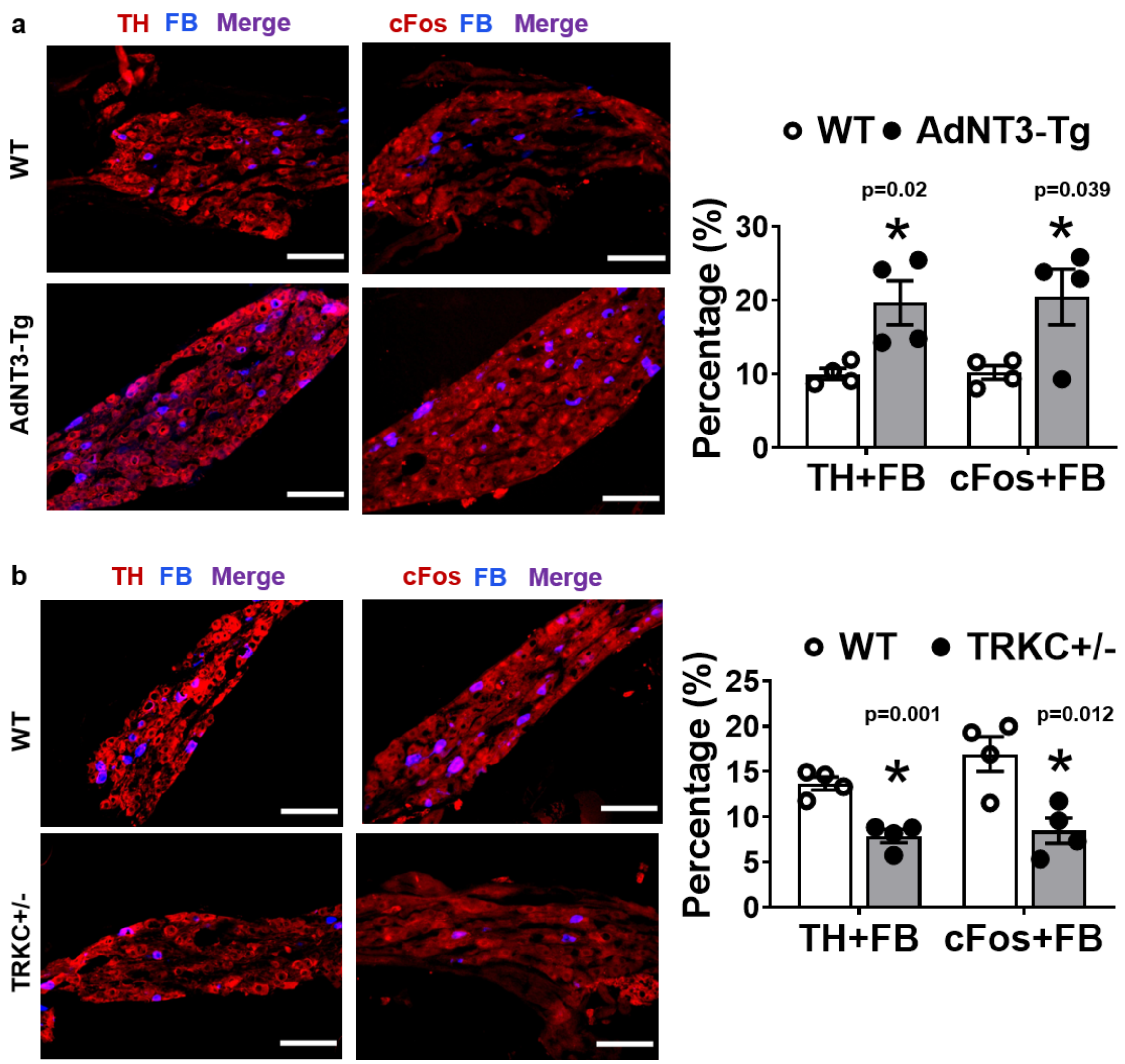

Supplemental Figure 15. (**a**) Representative images (left panel, from 4 replicate animals/group, scale bar=75μm) and quantitation (right panel, n=4/group, *indicates statistical significance between WT and AdNT3-Tg with unpaired two-tailed t-test) of TH/FB and cFos/FB labeling in sympathetic ganglia at lumbar L1 level in WT and AdNT3-Tg mice after a 7-day cold challenge.

(**b**) Representative images (left panel, from 4 replicate animals/group, scale bar=75μm) and quantitation (right panel, n=4/group, *indicates statistical significance between WT and TRKC+/- with unpaired two-tailed t-test) of TH/FB and cFos/FB labeling in sympathetic ganglia at lumbar L2 level in WT and TRKC+/- mice after a 7-day cold challenge.

All data are expressed as mean ± SEM.

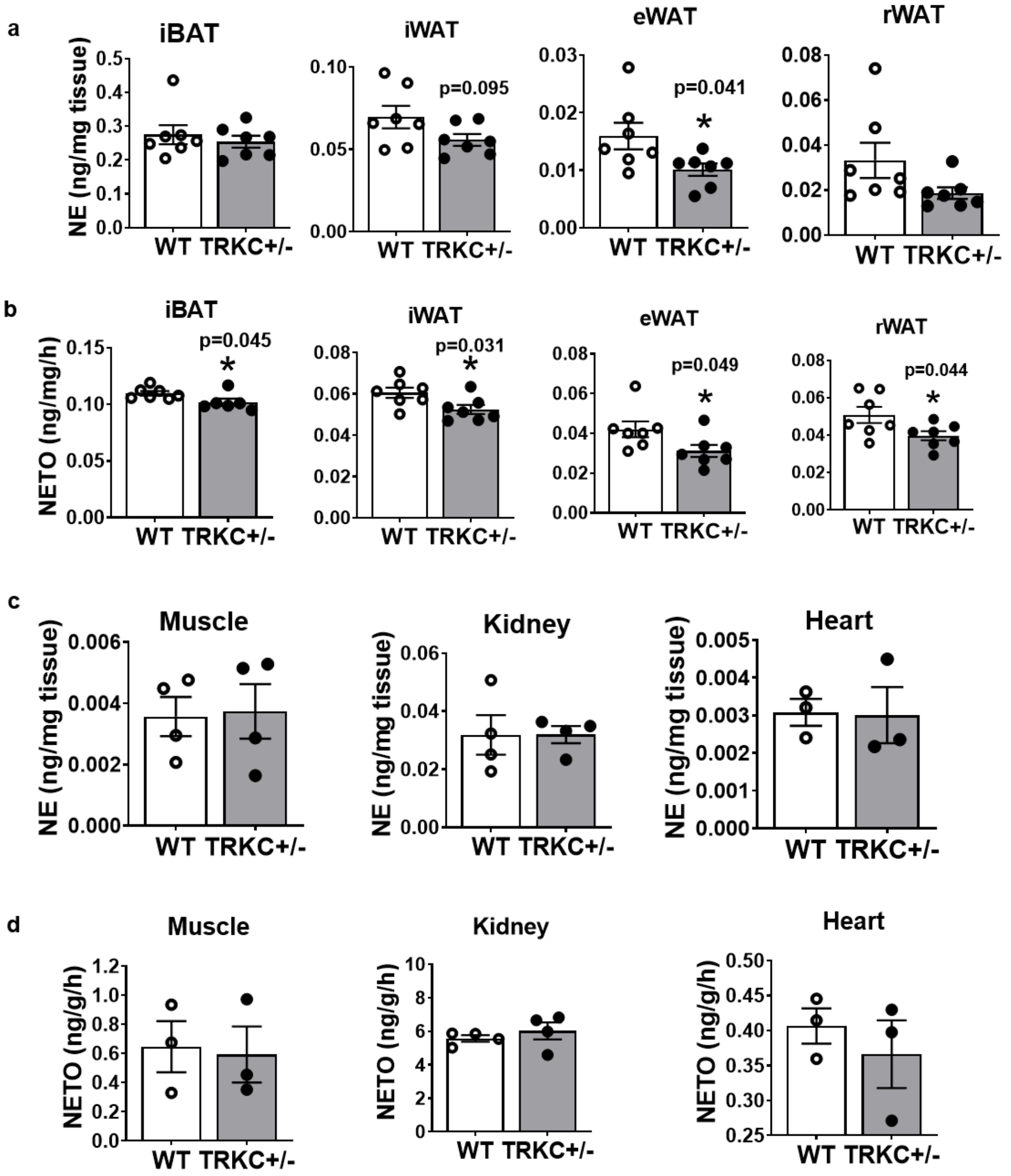

Supplemental Figure 16. Norepinephrine (NE) content and NE turnover (NETO) rate in adipose and other tissues of WT and TRKC+/- mice after a 16-hour cold challenge.

(**a**-**b**) Basal NE content (**a**, n=7/group, *indicates statistical significance between WT and TRKC+/- with unpaired two-tailed t-test) and NETO rate (**b**, for iBAT, WT=7, TRKC+/-=6; for iWAT, eWAT and rWAT, n=7/group; *indicates statistical significance between WT and TRKC+/- with unpaired two-tailed t-test) in iBAT, iWAT, eWAT and rWAT of WT and TRKC+/- mice after a 16-hour cold challenge.

(**c**-**d**) Basal NE content (**c**, muscle=4/group, kidney=4/group, heart=3/group) and NETO rate (**d**) in skeletal muscle, kidney and heart of WT and TRKC+/- mice after a 16-hour cold challenge (muscle=3/group, kidney n=4/group, heart=3/group).

All data are expressed as mean ± SEM.

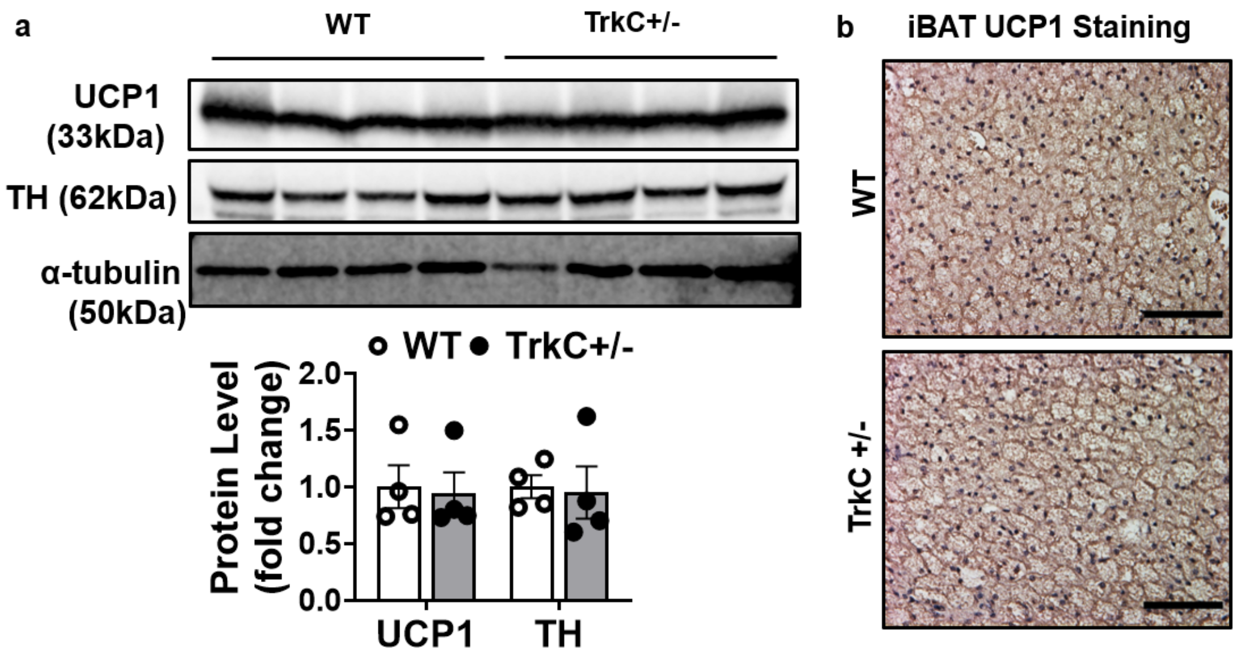

Supplemental Figure 17. UCP1 and TH protein levels (**a**, n=4/group) and representative UCP1 immunostaining in iBAT (**b**, from 3 replicate animals/group, scale bar=75μm) of 2-month-old WT and TRKC+/- mice subjected to a 7-day cold challenge.

All data are expressed as mean ± SEM.

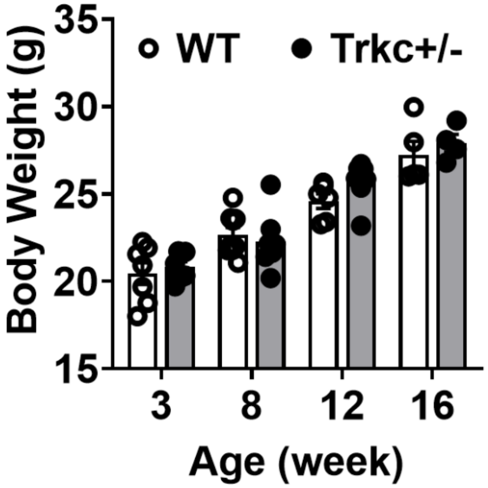

Supplemental Figure 18. Body weight in TRKC+/- and WT littermates fed regular chow diet housed at room temperature. (3 week: WT=7 TRKC+/-=8; 8 week: WT=9 TRKC+/-=7; 12 week: WT=6 TRKC+/-=8; 16week: WT=5 TRKC+/-=4).

All data are expressed as mean ± SEM.

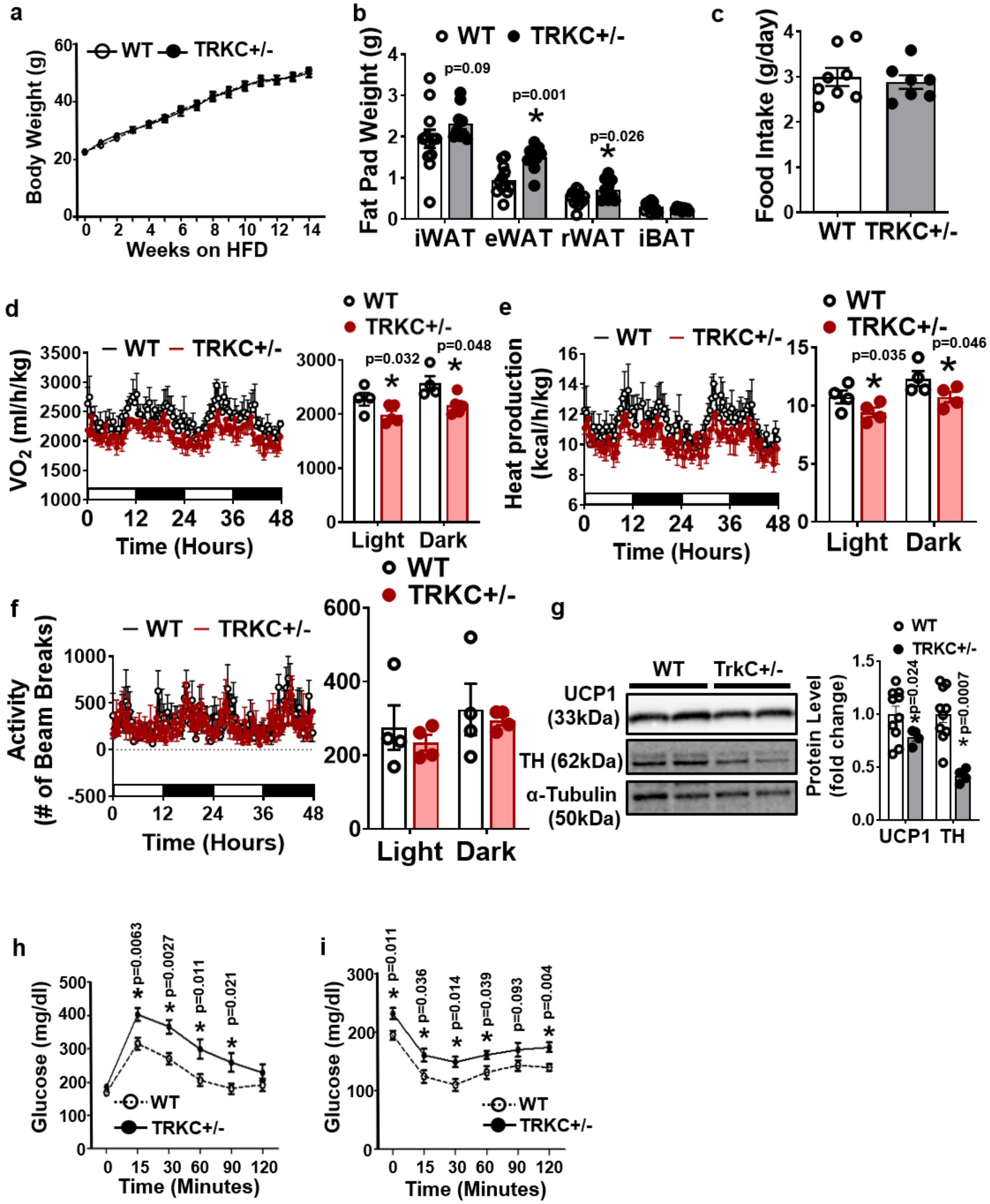

Supplemental Figure 19. Metabolic characterization of WT and TRKC+/- mice fed HFD at ambient room temperature (20-22°C).

(**a**-**i**) Body weight(**a**, WT n=9, TRKC+/- n=7), Fat pad mass (**b**, WT n=12, TRKC+/- n=9, *indicates statistical significance between WT and TRKC+/- with unpaired two-tailed t-test), Food intake (**c**, WT=8, TRKC+/-=7), Oxygen consumption(**d**, n=4/group, *indicates statistical significance

between WT and TRKC+/- with unpaired two-tailed t-test), Heat production (**e**, n=4/group, *indicates statistical significance between WT and TRKC+/- with unpaired two-tailed t-test), Locomotor activity (**f**, n=4/group), UCP1 and TH protein levels in iBAT (**g**, WT=10, TRKC+/-=4, *indicates statistical significance between WT and TRKC+/- with unpaired two-tailed t-test), GTT (**h**, WT=10, TRKC+/-=7, *indicates statistical significance between WT and TRKC+/- with unpaired two-tailed t-test) and ITT (**i**, WT n=8, TRKC+/- n=7, *indicates statistical significance between WT and TRKC+/- with unpaired two-tailed t-test) in WT and TRKC+/- mice fed a HFD at room temperature.

All data are expressed as mean ± SEM.

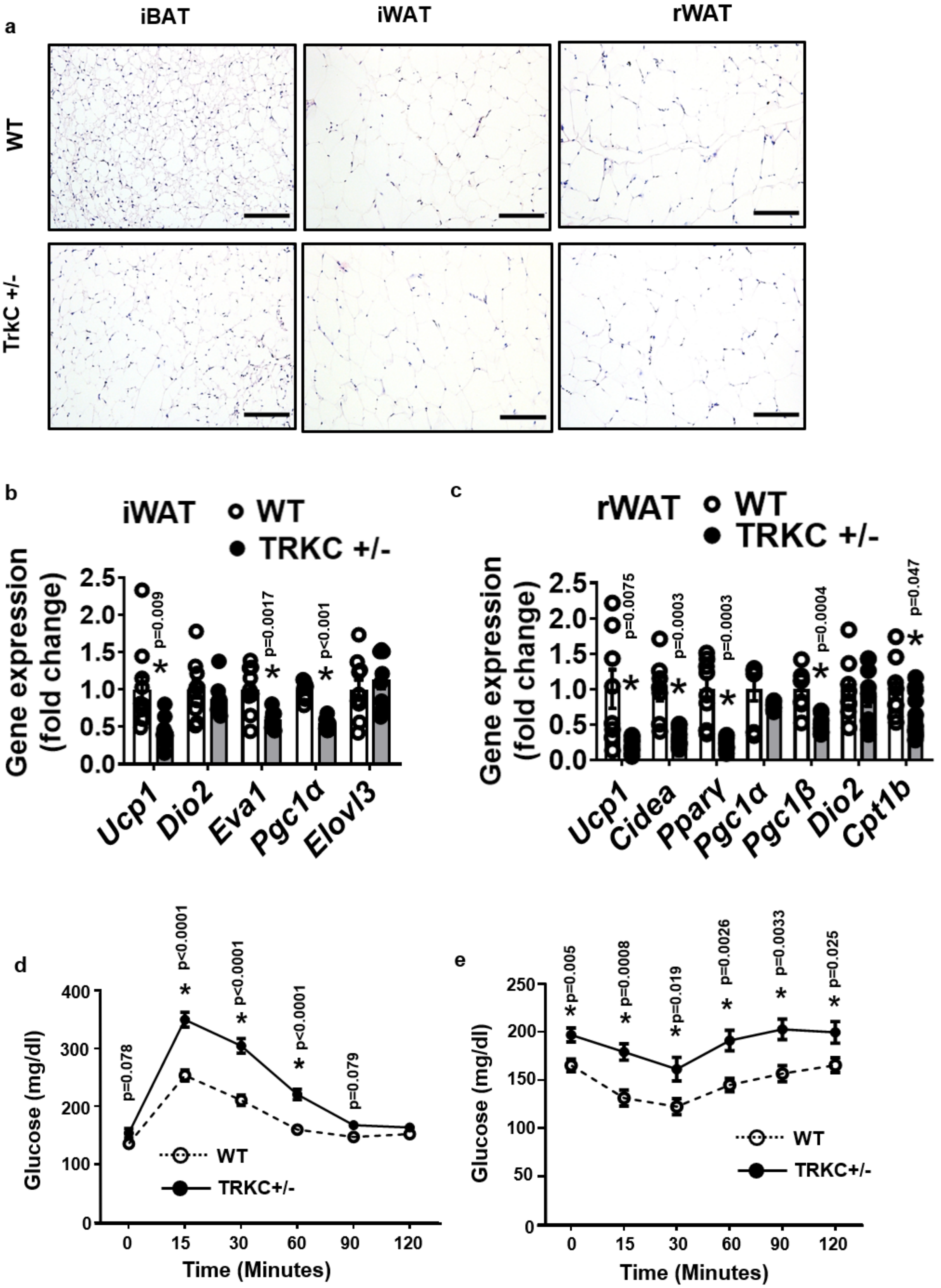
a
iBAT
iWAT
rWAT
WT
TrkC +/-
b
iWAT
WT
TRKC +/-
Gene expression (fold change)
2.5
2.0
1.5
1.0
0.5
0.0
p=0.009
p=0.0017
p<0.001
*
Ucp1
Dio2
Eva1
Pgc1α
Elovl3
c
rWAT
WT
TRKC +/-
Gene expression (fold change)
2.5
2.0
1.5
1.0
0.5
0.0
p=0.0075
p=0.0003
p=0.0003
p=0.0004
p=0.047
*
Ucp1
Cidea
Pparγ
Pgc1α
Pgc1β
Dio2
Cpt1b
d
Glucose (mg/dl)
400
300
200
100
0
p=0.078
p<0.0001
p<0.0001
p<0.0001
p=0.079
*
WT
TRKC+/-
0
15
30
60
90
120
Time (Minutes)
e
Glucose (mg/dl)
250
200
150
100
50
0
p=0.005
p=0.0008
p=0.019
p=0.0026
p=0.0033
p=0.025
*
WT
TRKC+/-
0
15
30
60
90
120
Time (Minutes)

Supplemental Figure 20. Metabolic characterization of WT and TRKC+/- mice fed HFD at thermoneutrality (30°C).

(**a**-**e**) Representative H&E staining of iBAT, iWAT and rWAT (**a**, from 3 replicate animals/group, scale bars for iBAT=75μm, for iWAT and rWAT=150μm), *Ucp1* and other thermogenic gene expression in iWAT (**b**, WT=10, TRKC+/-=9, *indicates statistical significance between WT and TRKC+/- with unpaired two-tailed t-test) and rWAT (**c**, WT n=8, for TRKC+/-, n=9 for Ucp1, Cidea, Dio2 and Cpt1β, n=8 for Pparγ and Pgc1β, and n=7 for Pgc1α. *indicates statistical significance between WT and TRKC+/- with unpaired two-tailed t-test), GTT (**d**, WT n=14, TRKC+/- n=9, *indicates statistical significance between WT and TRKC+/- with unpaired two-tailed t-test) and ITT (**e**, WT n=15, TRKC+/- n=9, *indicates statistical significance between WT and TRKC+/- with unpaired two-tailed t-test) in WT and TRKC+/- mice fed a HFD at thermoneutrality (30°C).

All data are expressed as mean ± SEM.

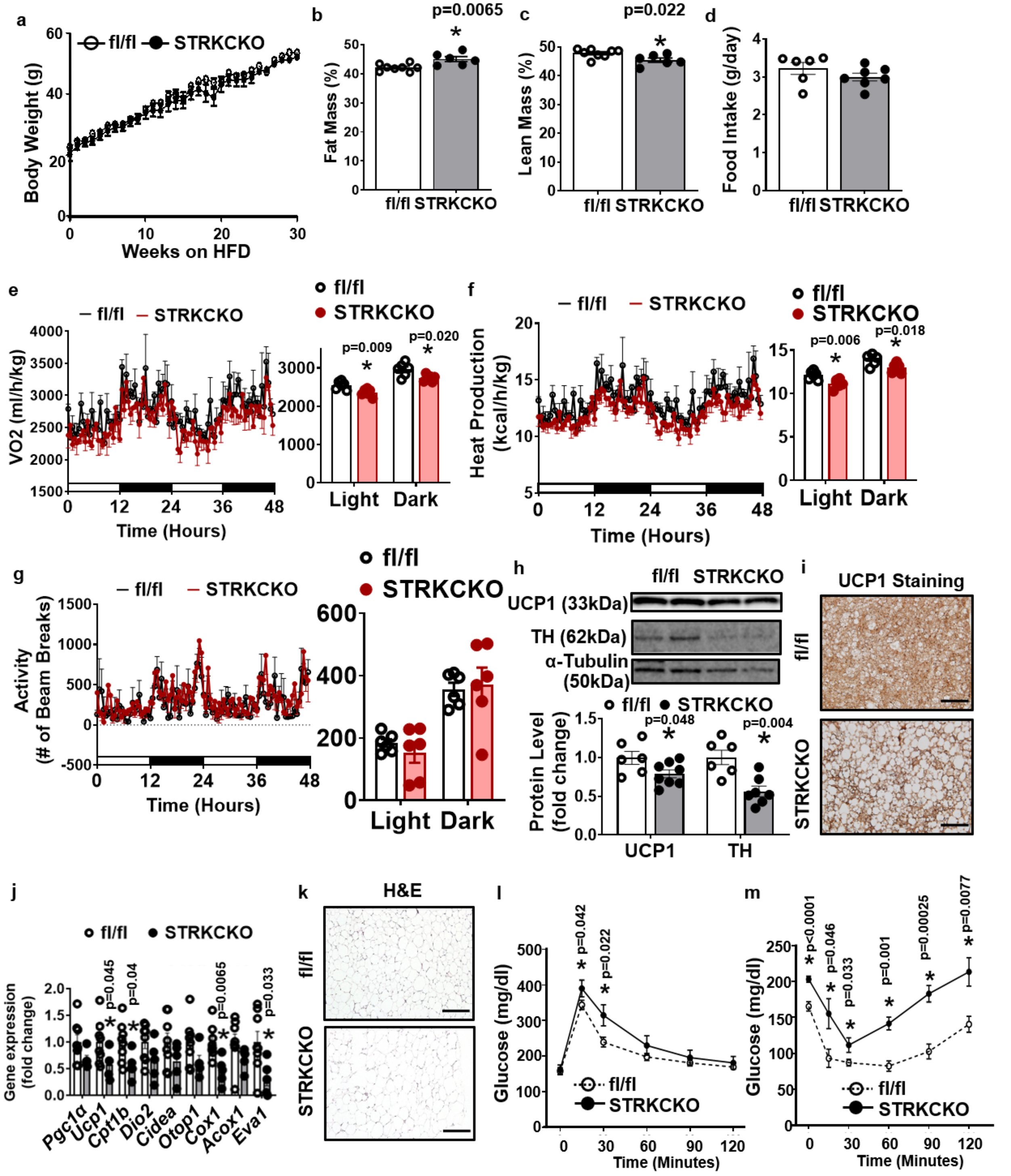

Supplemental Figure 21. Metabolic characterization of fl/fl and STRKCKO mice fed HFD at ambient room temperature (20-22°C).

(**a**-**m**) Body weight (**a**, fl/fl n=8, STRKCKO n=5), Fat mass (**b**, fl/fl n=8, STRKCKO n=6, *indicates statistical significance between fl/fl and STRKCKO with unpaired two-tailed t-test), Lean mass (**c**, fl/fl n=8, STRKCKO n=6), Food intake(**d**, fl/fl n=6, STRKCKO n=7), Oxygen consumption(**e**, n=6/group, *indicates statistical significance between fl/fl and STRKCKO with unpaired two-tailed t-test), Heat production (**f**, n=6/group, *indicates statistical significance between fl/fl and STRKCKO with unpaired two-tailed t-test), Locomotor activity (**g**, n=6/group), UCP1 and TH protein levels in iBAT (**h**, fl/fl n=6, STRKCKO n=8, *indicates statistical significance between fl/fl and STRKCKO with unpaired two-tailed t-test), Representative UCP1 immunostaining in iBAT (**i**, from 3 replicate animals/group, scale bar=75μm), *Ucp1* and other thermogenic gene expression in iWAT (**j,** fl/fl n=8, STRKCKO n=5, *indicates statistical significance between fl/fl and STRKCKO with unpaired two-tailed t-test), Representative H&E staining in iWAT (**k**, from 3 replicate animals/group, scale bar=150μm), GTT (**l**, fl/fl n=7, STRKCKO n=6, *indicates statistical significance between fl/fl and STRKCKO with unpaired two-tailed t-test) and ITT (**m**, fl/fl n=7, STRKCKO n=6, *indicates statistical significance between fl/fl and STRKCKO with unpaired two-tailed t-test) in fl/fl and STRKCKO mice fed a HFD at room temperature.

All data are expressed as mean ± SEM.
