# Supplementary material for: Adipose tissue-derived neurotrophic factor 3 regulates sympathetic innervation and thermogenesis in adipose tissue
Source: Nat Commun. 2021 Sep 10;12:5362. doi: 10.1038/s41467-021-25766-2 (PMC8433218; doi:10.1038/s41467-021-25766-2)
Supplement: Supplementary file 3 — Reporting Summary [file 41467_2021_25766_MOESM3_ESM.pdf]

Corresponding author(s): Bingzhong Xue
Last updated by author(s): 7/15/2021

# Reporting Summary

Nature Research wishes to improve the reproducibility of the work that we publish. This form provides structure for consistency and transparency in reporting. For further information on Nature Research policies, see our Editorial Policies and the Editorial Policy Checklist.

## Statistics

For all statistical analyses, confirm that the following items are present in the figure legend, table legend, main text, or Methods section.

| n/a | Confirmed | |
|---|---|---|
| ☐ | ☒ | The exact sample size (*n*) for each experimental group/condition, given as a discrete number and unit of measurement |
| ☐ | ☒ | A statement on whether measurements were taken from distinct samples or whether the same sample was measured repeatedly |
| ☐ | ☒ | The statistical test(s) used AND whether they are one- or two-sided<br>*Only common tests should be described solely by name; describe more complex techniques in the Methods section.* |
| ☒ | ☐ | A description of all covariates tested |
| ☒ | ☐ | A description of any assumptions or corrections, such as tests of normality and adjustment for multiple comparisons |
| ☐ | ☒ | A full description of the statistical parameters including central tendency (e.g. means) or other basic estimates (e.g. regression coefficient) AND variation (e.g. standard deviation) or associated estimates of uncertainty (e.g. confidence intervals) |
| ☐ | ☒ | For null hypothesis testing, the test statistic (e.g. $F$, $t$, $r$) with confidence intervals, effect sizes, degrees of freedom and $P$ value noted<br>*Give P values as exact values whenever suitable.* |
| ☒ | ☐ | For Bayesian analysis, information on the choice of priors and Markov chain Monte Carlo settings |
| ☒ | ☐ | For hierarchical and complex designs, identification of the appropriate level for tests and full reporting of outcomes |
| ☒ | ☐ | Estimates of effect sizes (e.g. Cohen's *d*, Pearson's *r*), indicating how they were calculated |

*Our web collection on statistics for biologists contains articles on many of the points above.*

## Software and code

Policy information about availability of computer code

| | |
|---|---|
| Data collection | 1. The expression of genes of interest was measured by a one-step quantitative RT-PCR with TaqMan Universal PCR Master Mix reagents (ThermoFisher Scientific, Waltham, MA) using an Applied Biosystems QuantStudio 3 real-time PCR system (ThermoFisher Scientific).<br>2. Histological and IHC images were captured using an Olympus DP73 photomicroscope or Zeiss 710 NLO Laser Scanning Confocal Microscope.<br>3. Immunoblotting was visualized using a Li-COR Imager System (Li-COR Biosciences, Lincoln, NE).<br>4. Tissue NE content was measured by HPLC.<br>5.For bioinformatics analysis, raw reads were filtered to remove adaptor sequences and low-quality data using SOAPnuke (v1.5.2, https://github.com/BGI-flexlab/SOAPnuke) and mapped to reference sequences (University of California Santa Cruz Mouse Genome Browser mm9 Assembly) using Hierarchical Indexing for Spliced Alignment of Transcripts (HISAT2, v2.0.4, http://www.ccb.jhu.edu/software/hisat/index.shtml). |
| Data analysis | Western blotting quantification was analyzed using Li-COR Image Studio (v2.1). Histological and IHC images were analyzed using Olympus CellSens (v1.6), Image J (v1.5.2), Neuron J (v1.4.3), Imaris Image Analysis Software (v9.5.1). All graphs were made with GraphPad Prism (v9.1.2). All statistical analysis was done with SPSS (v27.0.1.0). |

For manuscripts utilizing custom algorithms or software that are central to the research but not yet described in published literature, software must be made available to editors and reviewers. We strongly encourage code deposition in a community repository (e.g. GitHub). See the Nature Research guidelines for submitting code & software for further information.

## Data

Policy information about availability of data

All manuscripts must include a data availability statement. This statement should provide the following information, where applicable:

- Accession codes, unique identifiers, or web links for publicly available datasets
- A list of figures that have associated raw data
- A description of any restrictions on data availability

The RNAseq data have been deposited to Gene Expression Omnibus (GEO) database with the accession code GSE173503. All data generated in this study are available and reported. A source data file is included with the paper.

# Field-specific reporting

Please select the one below that is the best fit for your research. If you are not sure, read the appropriate sections before making your selection.

☒ Life sciences ☐ Behavioural & social sciences ☐ Ecological, evolutionary & environmental sciences

For a reference copy of the document with all sections, see nature.com/documents/nr-reporting-summary-flat.pdf

# Life sciences study design

All studies must disclose on these points even when the disclosure is negative.

| | |
|---|---|
| Sample size | The experimental sample sizes were estimated based on our previous published data that are sufficient to detect statistical differences among the groups (e.g. Li et al, J. Biol. Chem. VOL. 291, NO. 9, pp. 4523–4536, February 26, 2016; Cui et al, Physiol Rep, 4 (10), 2016, e12799). |
| Data exclusions | Most of the data were included for analysis. Only exception is, in real-time RT-PCR measurement, CT value for some samples were not detected due to experimental error and these samples were excluded from analysis. This includes one sample for Tgm1 and one sample for Pgm1 in Figure 7B, one sample for Pgc1b and one sample for Fgf21 in Suppl. Figure 10H, and one sample for Pparg, one sample for Pgc1b and two samples for Pgc1a in Suppl. Figure 20C. |
| Replication | A replication of 3 to 14 animals each group were used for animal studies, gene expression, immunobloting and histology analysis. For primary sympathetic neuronal culture, between 40 and 168 neurons each group were analyzed. The results were the same. |
| Randomization | For all experiments, animals with the same genotypes were randomly assigned into control and experimental groups. |
| Blinding | We applied blinding to immunohistochemistry sample analysis. For other experiments, blinding is not necessary because all groups including animals and cell experiments were treated the same way. |

# Reporting for specific materials, systems and methods

We require information from authors about some types of materials, experimental systems and methods used in many studies. Here, indicate whether each material, system or method listed is relevant to your study. If you are not sure if a list item applies to your research, read the appropriate section before selecting a response.

### Materials & experimental systems

| n/a | Involved in the study | |
|---|---|---|
| ☐ | ☒ | Antibodies |
| ☒ | ☐ | Eukaryotic cell lines |
| ☒ | ☐ | Palaeontology and archaeology |
| ☐ | ☒ | Animals and other organisms |
| ☒ | ☐ | Human research participants |
| ☒ | ☐ | Clinical data |
| ☒ | ☐ | Dual use research of concern |

### Methods

| n/a | Involved in the study | |
|---|---|---|
| ☒ | ☐ | ChIP-seq |
| ☒ | ☐ | Flow cytometry |
| ☒ | ☐ | MRI-based neuroimaging |

## Antibodies

| | |
|---|---|
| Antibodies used | Primary antibodies: UCP1 for western ABCAM ab23841, UCP1 for IHC ABCAM ab10983, TH Millipore AB152, NTF3 R&D AF-267-NA, α-tubulin Advanced BioChemicals ABCENT4777, cFos Millipore ABE457, GFP aves labs GFP1010, Anti-Beta III Tubulin Antibody, Alexa Fluor® 488 Conjugate Millipore AB15708A4, pHSL, Cell Signaling Technology 4126S, HSL, Cell Signaling Technology 4107S.<br><br>Secondary antibodies: Cy™3 AffiniPure Donkey Anti-Rabbit IgG (H+L) Jackson Immuoresearch 711-165-152, Alexa Fluor® 488 AffiniPure Donkey Anti-Chicken IgY (IgG) (H+L) Jackson Immuoresearch 703-545-155, Alexa Fluor 680 Goat Anti-Rabbit IgG (H / L), |

highly cross-adsorbed, Invitrogene A21109, Donkey anti-Goat IgG (H+L) Cross-Adsorbed Secondary Antibody, Alexa Fluor 680, Invitrogen A21084, Biotin-SP (long spacer) AffiniPure Donkey Anti-Rabbit IgG (H+L) Jackson Immuoresearch 711-065-152

Validation

These antibodies were used as recommended by the manufacturer and as cited extensively in previous publications. We have also tested different concentrations of each antibody and used the optimized concentration for each antibody. We further ensured that signals in Western Blots were present at the expected size for each target The relevant information for each primary antibody, from the manufacturer and online databases, is as follows:

UCP1 for western ABCAM ab23841, Rabbit polyclonal to UCP1. Tested by the manufacturer for detecting UCP1 in mouse, rat and dog by western blotting (WB) and immunohistochemistryPolyclonal Goat IgG (IHC), using mouse and rat brown adipose tissue as positive controls in WB. Ninety-nine Citations from the CiteAb database (https://www.citeab.com/antibodies/758073-ab23841-anti-ucp1-antibody?des=641734b5b4769e80).

UCP1 for IHC ABCAM ab10983, Rabbit polyclonal to UCP1, tested by the manufacturer to detect UCP1 in Mouse, Rat, Spermophilus tridecemlineatus using WB, IHC and immunocytochemistry (ICC). Four hundred and thirty-eight citations from the CiteAb database (https://www.citeab.com/antibodies/758072-ab10983-anti-ucp1-antibody?des=9095b59013736f21).

TH Millipore AB152, Rabbit polyclonal antibody, detects level of TH and has been published and validated for use in ELISA, immunofluorescence (IF), IHC, immunoprecipitation (IP) and WB. It is routinely evaluated by Western Blot on PC12 lysates by the manufacturer. 1065 citations from the CiteAb database (https://www.citeab.com/antibodies/222941-ab152-anti-tyrosine-hydroxylase-antibody?des=8514859c14cb8a02).

NTF3 R&D AF-267-NA, Polyclonal Goat IgG, Recommended by manfacturer to detect NTF3 by WB, IHC and in neutralization studies. Four citations from CiteAb database (https://www.citeab.com/antibodies/689271-af-267-na-nt-3-antibody?des=a7f1775e2235e089).

α-tubulin Advanced BioChemicals ABCENT4777, rabbit polyclonal antibody, detects α-tubulin from human, mouse and rat by WB, IHC, IF and ELISA.

cFos Millipore ABE457, rabbit polyclonal antibody. Evaluated by manfacturer by Western Blot in PMA(TPA) treated HeLa cell lysate. Recommended by the manufacturer to detect cFos by WB and IHC. Seven-six citations from CiteAb database (https://www.citeab.com/antibodies/1474449-abe457-anti-c-fos-antibody?des=59448cbafc416338).

GFP aves labs GFP1010, Chicken polyclonal antibody. Recommended by the manufacturer to detect GFP by ELISA, ICC, IHC, WB. From manufacturer's notes: Antibodies were analyzed by western blot analysis (1:5000 dilution) and immunohistochemistry (1:500 dilution) using transgenic mice expressing the GFP gene product. One hundred and sixteen citations from CiteAb database (https://www.citeab.com/antibodies/575207-gfp-1010-green-fluorescent-protein-1-0-mg?des=708459386bbbe89e).

Anti-Beta III Tubulin Antibody, Alexa Fluor® 488 Conjugate Millipore AB15708A4, rabbit polyclonal antibody. Recommended by the manufacturer to detect beta III Tubulin by IHC and ICC. Has been evaluated by the manufacturer by Immunocytochemistry in rat E18 cortex cells. Nine citations from CiteAb database (https://www.citeab.com/antibodies/1475014-ab15708a4-anti-beta-iii-tubulin-antibody-alexa-fluo?des=d650ee6554e7b12e).

pHSL, Cell Signaling Technology 4126S, recommended by the manufacturer to detect phosphorylated HSL at ser660 by WB. 191 citation from CiteAb database (https://www.citeab.com/antibodies/654213-4126-phospho-hsl-ser660-antibody?des=340bd2853a512cf6).

HSL, Cell Signaling Technology 4107S. Recommended by the manufactuer to detect endogenous total HSL by WB. 309 citations from CiteAb database (https://www.citeab.com/antibodies/123899-4107-hsl-antibody?des=c217aa1e3de30ca5).

Cy™3 AffiniPure Donkey Anti-Rabbit IgG (H+L) Jackson Immuoresearch 711-165-152, from the manufacturer website, this secondary antibody has minimal cross reactivity with Bovine, Chicken, Goat, Guinea Pig, Syrian Hamster, Horse, Human, Mouse, Rat, Sheep Serum Proteins. 1003 citations from CiteAb database: https://www.citeab.com/antibodies/2036162-711-165-152-cy3-affinipure-donkey-anti-rabbit-igg-h?des=fe83f7d682eb735d.

Alexa Fluor® 488 AffiniPure Donkey Anti-Chicken IgY (IgG) (H+L) Jackson Immuoresearch 703-545-155, from the manufacturer website, this secondary antibody has minimal cross reactivity with Bovine, Goat, Guinea Pig, Syrian Hamster, Horse, Human, Mouse, Rabbit, Rat, Sheep Serum Proteins. 365 citations from CiteAb database: https://www.citeab.com/antibodies/2034669-703-545-155-alexa-fluor-488-affinipure-donkey-anti-c?des=8beb9467dd8f19ff.

Alexa Fluor 680 Goat Anti-Rabbit IgG (H ⁄ L), highly cross-adsorbed, Invitrogene A21109. According to manufacturer, to minimize cross-reactivity, these goat anti-rabbit IgG (H+L) whole secondary antibodies have been affinity purified and cross-adsorbed against bovine IgG, goat IgG, mouse IgG, rat IgG, and human IgG. Cross-adsorption or pre-adsorption is a purification step to increase specificity of the antibody resulting in higher sensitivity and less background staining. The secondary antibody solution is passed through a column matrix containing immobilized serum proteins from potentially cross-reactive species. Only the nonspecific-binding secondary antibodies are captured in the column, and the highly specific secondaries flow through. The benefits of this extra step are apparent in multiplexing/multicolor-staining experiments (e.g., flow cytometry) where there is potential cross-reactivity with other primary antibodies or in tissue/cell fluorescent staining experiments where there are may be the presence of endogenous immunoglobulins. 201 citations from CiteAb: https://www.citeab.com/antibodies/2401223-a-21109-goat-anti-rabbit-igg-h-l-highly-cross-adso?des=cb3719bfcb178f19.

Donkey anti-Goat IgG (H+L) Cross-Adsorbed Secondary Antibody, Alexa Fluor 680, Invitrogen A21084. This secondary antibody is designed for fluorescent Western blot detection on various near-infrared fluorescence instruments. This antibody can be used for multi-color and multiplexing detection when using other antibodies conjugated to compatible Alexa Fluor dyes and wavelengths. Other applications of this antibody include immunofluorescent and fluorescent imaging applications when using instrumentation with appropriate excitation and detection capabilities. 56 citations from CiteAb: https://www.citeab.com/antibodies/2401206-

a-21084-donkey-anti-goat-igg-h-l-cross-adsorbed-se?des=835135c6122e19ad.

Biotin-SP (long spacer) AffiniPure Donkey Anti-Rabbit IgG (H+L) Jackson Immuoresearch 711-065-152. From the manufacturer website, this secondary antibody has minimal cross reactivity with Bovine, Chicken, Goat, Guinea Pig, Syrian Hamster, Horse, Human, Mouse, Rat, Sheep Serum Proteins. 380 citations from CiteAb: https://www.citeab.com/antibodies/2035909-711-065-152-biotin-sp-affinipure-donkey-anti-rabbit?des=ed9de846d3ea1d0d.

## Animals and other organisms

Policy information about studies involving animals; ARRIVE guidelines recommended for reporting animal research

| | |
|---|---|
| Laboratory animals | C57BL/6J, TRKC+/- (Jackson Laboratory, #002481), TRKC-flox (Jackson Laboratory #022364), TH-Cre (Jackson Laboratory #008601), TRKC-Cre (MMRRC #000364-UCD),R26-stop-EYFP(Jackson Laboratory #006148), Flp mice Jackson Laboratory, Stock No. 009086), Adiponectine-Cre (Jackson Laboratory #010803), Adiponectin-NTF3-TG (made in the lab), Adiponectin-NTF3-KI (made in the lab), and Rosa-NTF3fl/+ mice (made in the lab). For all these strains, male mice from around 3 weeks to up to 36 weeks old were used in the studies. Most animals were maintained in a temperature controlled room (20°C-22°C) with humidity ranging from 30-70%, and with a 12-hour light/dark cycles (0700-1900 light). For experiments with thermoneutral condition, animals were housed at 30 °C with the same humidity level and 12-hour light/dark cycle. |
| Wild animals | No wild animals were used in this study. |
| Field-collected samples | No field-collected samples were used in this study. |
| Ethics oversight | All animal procedures were approved by the Institutional Animal Care and Use Committee of Georgia State University. |

Note that full information on the approval of the study protocol must also be provided in the manuscript.
